# Classification of CRISPR/Cas system and its application in tomato breeding

**DOI:** 10.1007/s00122-021-03984-y

**Published:** 2022-01-01

**Authors:** Abira Chaudhuri, Koushik Halder, Asis Datta

**Affiliations:** National Institute of Plant Genome Research, Aruna Asaf Ali Marg, P.O. Box No. 10531, New Delhi, 110 067 India

## Abstract

Remarkable diversity in the domain of genome loci architecture, structure of effector complex, array of protein composition, mechanisms of adaptation along with difference in pre-crRNA processing and interference have led to a vast scope of detailed classification in bacterial and archaeal CRISPR/Cas systems, their intrinsic weapon of adaptive immunity. Two classes: Class 1 and Class 2, several types and subtypes have been identified so far. While the evolution of the effector complexes of Class 2 is assigned solely to mobile genetic elements, the origin of Class 1 effector molecules is still in a haze. Majority of the types target DNA except type VI, which have been found to target RNA exclusively. Cas9, the single effector protein, has been the primary focus of CRISPR-mediated genome editing revolution and is an integral part of Class 2 (type II) system. The present review focuses on the different CRISPR types in depth and the application of CRISPR/Cas9 for epigenome modification, targeted base editing and improving traits such as abiotic and biotic stress tolerance, yield and nutritional aspects of tomato breeding.

## Introduction

Genetic diversity is a potential resource for a broad range of genetic research and trait improvement in plants. The gradual evolution in the plant breeding technologies and expansion of its possibilities is much required to cope up with the incessantly increasing needs of man (Xiong et al. 2015). With the aim of creating new varieties, breeders have developed novel methods to introduce heritable mutations into plant genomes. In the recent past, various mutagens like chemical compounds and irradiation were used to generate large pools of genetic variation in traditional breeding. Like all methods, these too have several drawbacks, such as the non-specific nature of the generated mutations, simultaneous mutation of a large amount of nucleotides followed by deletion, duplication and rearrangement of lengthy genomic fragments (Hartwell et al. 2018), thus making the identification of the mutations a laborious process. Also the random mutagenesis methods usually prove to be less effective to improve traits in polyploid crops, because of their extreme genetic redundancy (Mao et al. 2019).

Although traditional breeding allowed the selection of unique crops with improved traits, enriched qualities and extended shelf life coupled with long breeding cycles yet lack of precision in hybridization, high ratio of heterozygosities along with low frequencies of the desirable mutation have led to the development of less/moderate resource-demanding technologies. Recombinant DNA technology has proved to be versatile since it can overcome the incompatibility issues between two species by the integration of foreign genes into the target plant genomes to get the desired traits (Genetically modified crops, GM crops). So far, GM crops constitute a significant proportion of the diet among the populations of America, Australia, China and other developing nations. However, several countries including India, oppose the use of GM crops for human consumption on the ground that it might pose risk to human health and environment. However, until now no long-term scientific study have been able to gather evidence that GM crops have profound adverse effect on human health and environment as compared to the existing plant breeding technologies (Council 2013). So this so-called debate seems to be far-fetched from the knowledge based upon solid scientific research and centered solely upon the moral traditions and political environment. Political hesitation regarding genetically modified organisms (GMOs) can easily be pointed out with the decision by seventeen European countries in 2015 to opt out of the possible commercialization of genetically modified food and fiber (Bonny 2003). The major concerns shown regarding GMOs is that they have been generated by introduction of a transgene into the host genome and the expression cassette (generally carrying viral promoter/terminator) often stayed there within the system (Li et al. 2019a). From that context, next-generation genome editing tools such as clustered regularly interspaced short palindromic repeats/Cas9 (CRISPR/Cas9) gain importance, as they are capable of introducing small insertion or deletion of nucleotides within the target gene itself, by using non-homologous end joining repair mechanism (NHEJ). Such genetic alteration does not involve the addition of any transgene and it is very much similar like the natural variation (Huang et al. 2016; Globus and Qimron 2018). Moreover, the expression cassette can be discarded from the progeny by genetic segregation or it is not at all required in case of ribonucleoprotein complex approach. Therefore, genome edited crops are very much different from GM crops in terms of their genetic properties and could be considered to have minimal/no risk to human life and its environment (Li et al. 2019a; Schulman et al. 2020). Spreading of more awareness and active cooperation will be required from both the scientific community and government agencies in order to gain public acceptance of the genome edited crops.

## CRISPR/CAS system

**Fig. 1:**
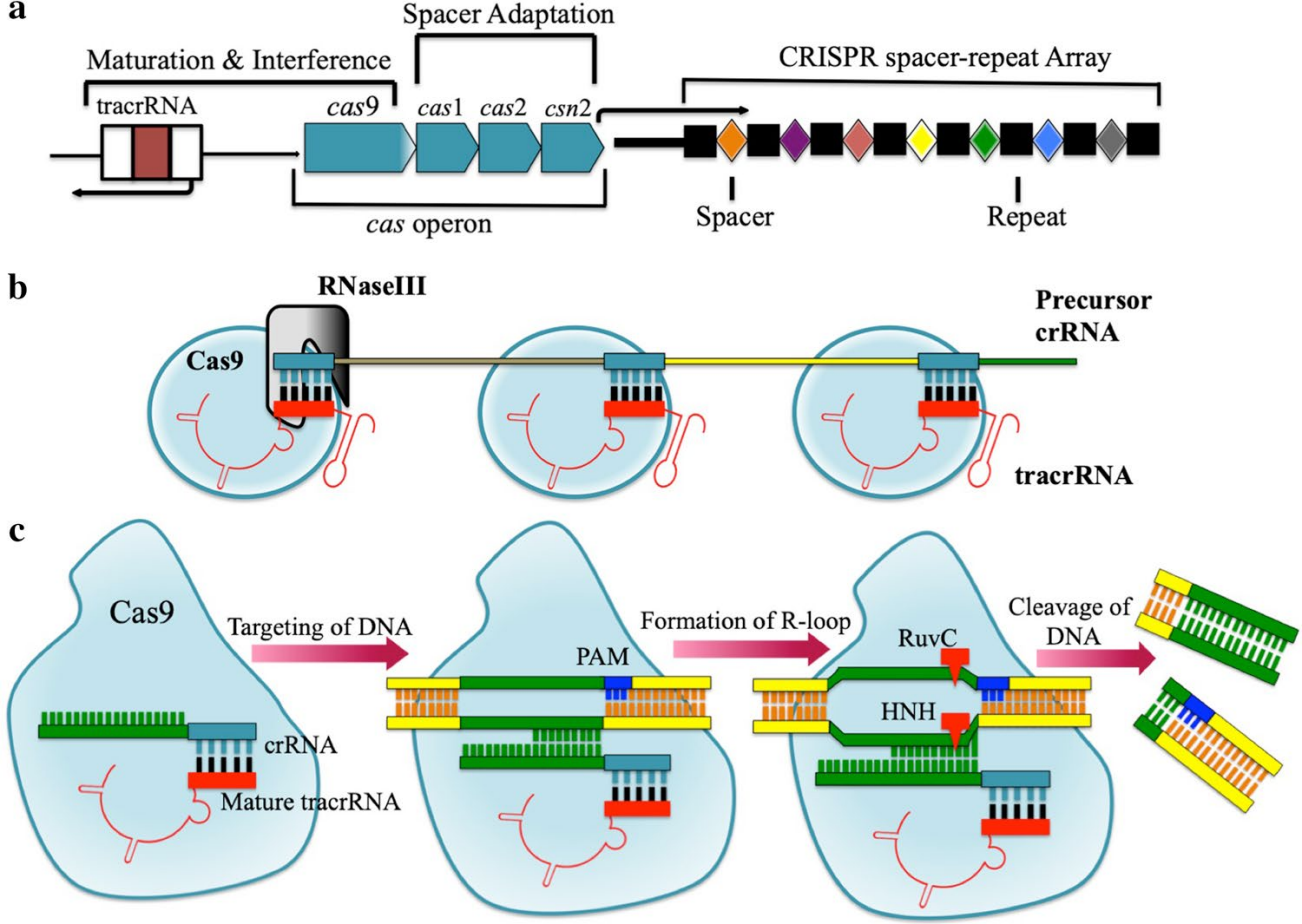
Type IIA CRISPR/Cas system in *Streptococcus pyogenes.* **a** CRISPR locus in genome: CRISPR locus consists of CRISPR repeats and spacer array along with tracrRNA and Cas operon. New spacers from the invading organism are incorporated into this CRISPR array (adaptation). **b** crRNA:tracrRNA co-maturation and complex formation with Cas9: pre-crRNA becomes matured crRNA with the help of tracrRNA along with Csn1 and Rnase III. Matured crRNA with Cas9 gets incorporated into ribonucleoprotein complexes which start scanning for nucleic acids complimentary to the sequence coded by crRNA (maturartion). **c** RNA-guided Cas9-mediated cleavage of targeted DNA: Upon recognition, complementary foreign sequences are cleaved by this Cas protein complex (Interference)

In recent years, the RNA-programmable CRISPR/Cas9 technology of genome editing has caught the eyes of researchers and has traversed a long way in a very short period since it is an uncomplicated and effortless process. The field of biology (animal, plant and microbe) has undergone a massive transformation because of the immense potential of this powerful genome editing tool. CRISPR was first described by Japanese researchers in 1987 while working with *iap* genes in *Escherichia coli*, as a small stretch of highly homologous repeats interspaced with small spacers (Ishino et al. 1987)*.* Years later, in 2005, three independent studies proved that these spacer sequences are acquired from external mobile genetic elements such as virus or plasmids (Bolotin et al. 2005; Pourcel et al. 2005; Soria 2005). CRISPR was found out to be present in various other bacteria and archaea (Mojica et al. 2000) that serves primarily as bacterial adaptive defense mechanism (Makarova et al. 2006; Barrangou et al. 2007). The CRISPR loci consist of CRISPR repeats along with DNA-targeting spacers and a *cas* operon that contains all the Cas protein coding genes (Fig. 1a).

CRISPR/Cas-mediated adaptive immunity occurs in three different stages: i) new spacers from the invading organism are incorporated into the CRISPR array (Adaptation) (Barrangou et al. 2007), ii) precursor crRNAs (pre-crRNA) are transcribed from the CRISPR array which then undergoes maturation and becomes a set of CRISPR RNAs (crRNA) carrying a single targeted spacer flanked by repeat sequences. This maturation process is directed by trans-activating CRISPR RNA (tracrRNA) with the help of CRISPR-associated Csn1 protein and endogenous RNase III. These crRNAs along with Cas proteins get incorporated into ribonucleoprotein (RNP) complexes, which then start scanning for nucleic acid sequences that are complementary with the sequence coded by crRNA (Maturation) (Deltcheva et al. 2011; Jinek et al. 2012; Zhang et al. 2012; Reeks et al. 2013). iii) Upon recognition, foreign sequences are cleaved by crRNA-guided Cas protein complex (Interference) (Semenova et al. 2011; Doudna and Charpentier 2014; Burmistrz et al. 2020) (Fig. 1b, c).

### How it is unique from other traditional gene editing tools

Investigators have been using two complementary yet different approaches to elucidate the function of a particular gene. First is the forward genetics approach that depends on the observation of phenotypes, thereby trying and linking it to its genetic basis; and another one is the reverse genetics approach, which creates insertion/deletion to induce mutation in the gene itself, thereby observing the change in the phenotype. RNA interference (RNAi) is the reverse genetics approach where investigator must have the prior knowledge of the gene sequence to be worked on. Irrespective of having been used extensively, it has been reported that RNAi often produce hypomorphic phenotype, which means there is a potential chance of getting a partial loss-of-function phenotype after targeting the gene of interest (Heigwer et al. 2018). To overcome this situation investigators need a more robust and unique reverse genetics approach and that is where zinc finger nuclease (ZFN) and transcription activator-like effector nuclease (TALEN) come in. As opposed to RNAi, these processes can achieve a complete loss-of-function phenotype by using customizable DNA-binding domains (DBDs) for recognition and targeting. These DBDs along with nucleases are capable of introducing double-stranded breaks (DSBs) into the targeted gene sequence causing frameshift mutation. The TALEN activity need not be maintained in the target cell, as these mutations are permanent in nature, whereas in RNAi mechanism, significant loss of siRNAs can dramatically reduce the loss-of-function phenotype. On the other hand CRISPR/Cas9 system is the powerful genome editing tool that could be used both as a forward (Wang et al. 2014; Chen et al. 2015) and reverse genetics approach (Orack et al. 2015; Mianné et al. 2016). CRISPR interference (CRISPRi) has more profound knockdown effect (Gilbert et al. 2015) as opposed to RNAi. Probably the most striking difference between RNAi and Cas9 nuclease-mediated editing is that RNAi can induce transient knockdown of gene expression, whereas Cas9 nuclease can induce permanent damage in target DNA (Boettcher and McManus 2015). Compared to TALEN and CRISPR/Cas9, ZFNs are expensive to make, and considerable labor is required to construct these high-affinity DBDs. TALENs surpass this bottleneck since they need considerably fewer enhancements post-construction, but they do depend on the lengthy assembly process of the minute building blocks to generate artificial DNA-binding proteins. However, the lengthy process can be surpassed with the most recent CRISPR/Cas9 tool since it caters to a technically uncomplicated system of genome editing (Lozano-Juste and Cutler 2014). More than one gene editing technology has been used to target the same gene (Table 1) and CRISPR/Cas-mediated gene editing showed better results.

**Table 1:** List of genes that have been targeted by both CRISPR/Cas9 and other traditional gene silencing methods such as RNAi, virus-induced gene silencing (VIGS), TALENs and the comparative analysis of their effects in the mutant lines

Gene	Tool	Cultivar	Phenotypic change	No of Lines screened	Reference
*PMR4*	RNAi	Moneymaker	Mutants showed resistance against powdery mildew	8	Huibers et al. (2013)
	CRISPR/Cas9	Moneymaker	Mutants showed enhanced resistance to powdery mildew pathogen, followed by higher level of hypersensitive responses like cell death at sites of fungal infection	37	Santillán Martínez et al. (2020)
*MAPK3*	VIGS	Y19	Mutant lines showed susceptibility to TYLCV infection	3	Li et al. (2017)
	CRISPR/Cas9	Ailsa Craig	Mutant lines showed susceptibility to gray mold disease, enhanced accumulation of reactive oxygen species	2	Zhang et al. (2018)
*MYB (ANT1)*	TALENs	Micro-Tom	High anthocyanin level, indel frequency is 14.27%	1881 cotyledons transformed with BeYDV-TALENs 1193/1194 construct	Čermák et al. (2015)
	CRISPR/Cas9	Micro-Tom	High anthocyanin level, NHEJ-induced indel frequency is ~ 28%	216 and 218 cotyledons transformed with BeYDV-Cas9/gRNA1b and BeYDV-Cas9/gRNA7 constructs, respectively	Čermák et al. (2015)

### PAM-free CRISPR/Cas9 system

With the gradual evolution and advancement of the CRISPR/Cas technology, the modern researchers started manipulating this system according to the need of smooth working and eventually the possibility for relaxed protospacer adjacent motif (PAM) requirements emerged. The field has advanced by taking long strides targeting a purely PAM-free nuclease territory. PAM-free nucleases have been constructed through the strategies like natural ortholog mining and protein engineering (Collias and Beisel 2021). Genetic engineering of Cas9 from the human pathogen *Streptococcus pyogenes* (SpyCas9) has relaxed its PAM profile considerably to one of two bases at a particular position. Numerous other Cas9 nucleases along with modified Cas12a nucleases are in the pipeline of being engineered having distinct properties like smaller size, high thermostability, etc.

**Fig. 2:**
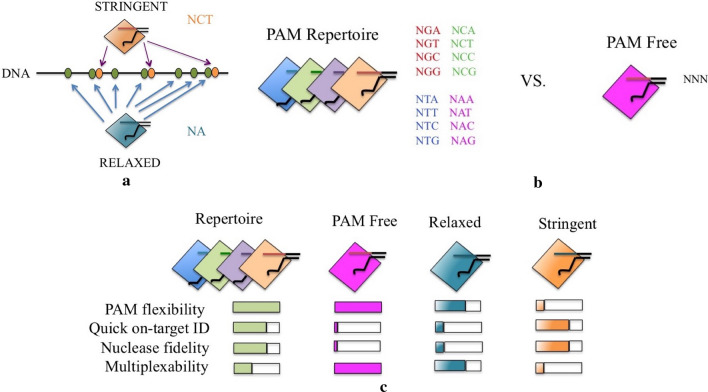
Development toward a PAM-free CRISPR/Cas system. **a** A comparative account depicting target accessibility by Cas nucleases having relaxed or stringent PAM necessity. (N, any base; C, cytosine; T, thymine; A, adenine). **b** A comparative account between a repertoire of nucleases and PAM-free nuclease. The former recognize every possible sequence together. The repertoire is a collection of four nucleases that read one letter at position two. (N, any nucleotide; A, adenine; G, guanine; T, thymine; C, cytosine). **c** A Comparative qualitative account between nuclease repertoire, PAM-free nuclease, PAM-relaxed nuclease and a PAM-stringent nuclease. The comparison is on the based on the capacity of targeting performance under different conditions. Greater performance of the associated nuclease is represented by a more filled bar. Nuclease fidelity is studied on the capacity of the nuclease to bypass nontarget sequence having even a minimal match with the guide sequence. Multiplex ability is assessed on the capacity of the nuclease to be put to use in targeting any random set of sequences

The major advantage of a PAM-free nuclease is the ability to target any sequence (Fig. 2a). The selection of sites would be simplified and flexible with preferably high on-target and low off-target activity, thus producing disruptive insertions and deletions, which are highly predictable (Chakrabarti et al. 2019). The placement of the base-editing window can also be arranged over the specific nucleotide directly. These flexible features act to be truly beneficial during multiplex editing since only one nuclease comes into full action and targets all the sequences. There are serious derogatory results as well of the PAM-free system (Fig. 2c). The guide RNAs (gRNAs) that are expressed from the DNA constructs would self-target the parent DNA immediately, ultimately leading to disastrous consequences. Another drawback of the PAM-free system is that a nuclease with no specific PAM will touch down on every sequence of that particular genome, thus taking a much longer time than required to finally find out its actual target and there would be increased risk of off-targeting (Fig. 2b). Apart from these potential drawbacks, this domain of PAM-free CRISPR/Cas system of genome editing is being taken seriously and constantly worked upon. The SpyCas9 group of nucleases have been engineered and made almost PAM-free, and the other remaining Cas nucleases like Cas9, Cas12a have much scope of relaxing their PAM recognition status and gradually move toward attaining a PAM-free status. To expedite the process of development for these above-mentioned nucleases, a combination of the procedures of ortholog mining and PAM engineering might prove to be highly effective and fruitful as already observed in the case of generating *Streptococcus canis* Cas9-Sc +  + and another high-fidelity mutant HiFi-Sc +  + (Chatterjee et al. 2020). Working on the not so well-characterized CRISPR/Cas types like type I and V could lead to major breakthrough by engineering type V–C nucleases, which have already been shown to recognize PAMs with a minimal of single base (Yan et al. 2019). An effective alternative to the entire issue of PAM-free nucleases in CRISPR/Cas system of genome editing is a nuclease repertoire (Fig. 5b), where the nuclease retains its property of recognition of a specific PAM sequence, that might be of a single base (e.g., NG) or a collection of bases (e.g., NAAA). This nuclease repertoire could be customized accommodating all the possible sequences, which would ultimately confer an overall PAM-free status to it. Since each nuclease retains its PAM recognition ability, it can bypass the shortfalls of the PAM-free system. Here the nucleases can be employed based on the required target on the basis of the flanking sequences. Hence, generation of a nuclease repertoire can be a potential solution by which any and every sequence can be targeted by CRISPR/Cas system.

### An efficient CRISPR toolkit for tomato breeding: Golden Gate

It has been proved now and again that CRISPR/Cas9 system of genome editing is the superpower in the domain of plant genome editing. The use of multi-single gRNAs (sgRNAs) to target single or multiple genes at a time has enabled in improving its gene-editing efficacy by leaps and bounds. An extremely handy and conveniently generated tool kit, which is used extensively in tomato breeding, is the Golden Gate Tool Kit. Engler et al. 2008, devised the Golden Gate cloning strategy. This strategy utilized the type IIS set of restriction enzymes that perform cuts far from their recognition sites. With neat and skillful design of cleavage sites, the two pieces cleaved by the above restriction enzymes can be joined together resulting in an end product minus the original restriction site. Thus, the above strategy (restriction–ligation) is instrumental in producing in one-pot and one-step highly pure and correct recombinant plasmids in minimal time (Engler et al. 2008). In a nutshell, this strategy allows fast and flexible assembly of genetic constructs and combination of diverse functional modules, respectively, for various applications (Čermák et al. 2017).

**Fig. 3:**
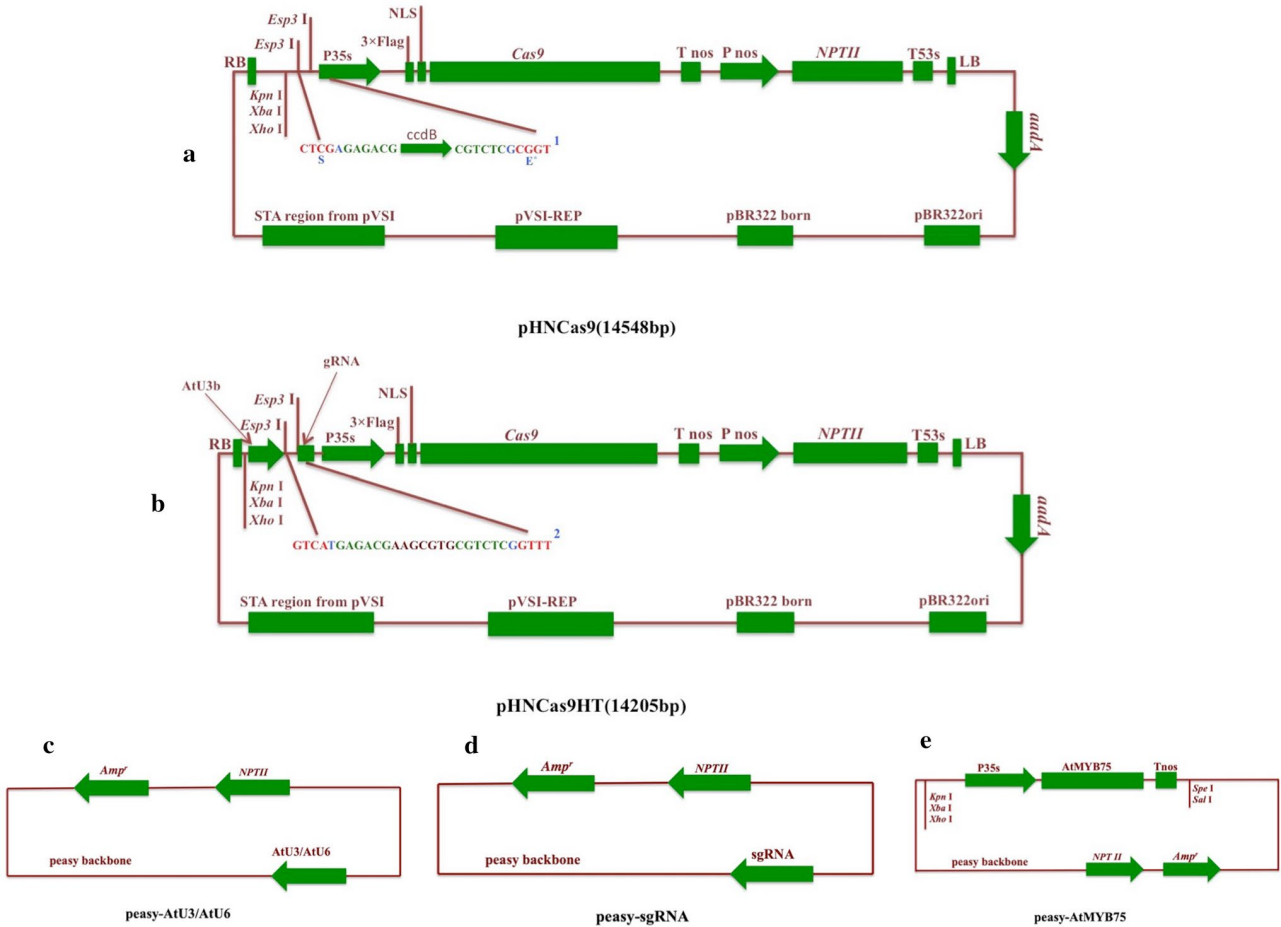
Golden gate: The efficient CRISPR tool kit. **a** Detailed structure of the binary vector pHNCas9, design based on pCAMBIA (Cambia, Canberra, Australia) vector backbone. (NPT II, antibiotic resistance marker neomycin phosphotransferase II; NLS, nuclear localization sequence; 1 and 2: Esp3 I restriction site colored in green, Golden Gate site Esp3 I cutting site colored in red; S, Golden Gate Site S links pHNCas9 binary vector with the TS’ site of the sgRNA expression cassettes. E′: Golden Gate Site E′ links the pHNCas9 binary vector with the TE site of the sgRNA expression cassettes. ccdB: the negative selectable marker ccdB gene. **b** Detailed structure of pHNCas9HT binary vector, design based on pHNCas9 vector. The sgRNA expression cassettes are regulated by AtU3b. Two Esp3 I restriction sites were put between sgRNA and AtU3d. **c** Overall and detailed structures of vectors pEASY-AtU3b, pEASY-AtU3d, pEasy-AtU6-1, pEASY-AtU6-26 and pEASY-AtU6-29. **d** Detailed structure of pEASY-sgRNA vectors. **e** Detailed structure of pEASY-OEAtMYB75 vectors

Tomato has proved to be a perfect example of a model plant where CRISPR/Cas system has been an efficient tool in creating new varieties without compromising the plant genome with foreign genes. Hu et al. 2019 have devised a highly innovative and flexible modular system in the domain of plant genome engineering for functional genomics in tomato and other potential food and cash crops. This research group applied standardized BioBrick technology to make this system more flexible for upgradation and accommodating toward novel expression elements. BioBrick technology had been described as a synthetic biology technology that works on the basis of same-tail restriction enzyme where BioBrick modules are assembled without break, keeping the BioBrick sites intact. Their (Hu et al. 2019) research approach was to develop a *Cas9* system comprising of two binary vectors pHNCas9 and pHNCas9HT (Fig. 3a, b). pHNCas9HT has the capacity to construct sgRNA expression cassettes bypassing the pivotal processes of PCR and direct *Agrobacterium tumifaciens* transformation. Golden Gate ligation strategy comes into the picture over here, where pHNCas9 vector is used for the assembly of myriad sgRNA expression cassettes in one-pot. The type II restriction endonucleases used here are *Esp3* I and *Bsa* I. They produce non-palindromic sticky end sites, which totally cuts off all possibilities of self-ligation and incompatible end ligation. So, this is a perfect method to link numerous DNA fragments in one-step and one-tube which can be simultaneously used for single site and multi-site editing. Apart from the two binary vectors the system provides with 5 separate Pol III-dependent promoters, sgRNA in pEASY cloning vector, 8 pairs of Bsa I-site primer along with a gene specific primer design aid. All the components together can make a perfect CRISPR tool kit (Fig. 3c–e).

In the experimental setup, a visual T-DNA marker was designed by utilizing a BioBrick. This T-DNA marker design used the overexpression of *AtMYB75/PAP1* (having 35S promoter) as a template. The genome editing systems possessing visual markers makes screening of transformants smooth and hassle-free since a different plant color is the indicator of whether the T-DNA marker with CRISPR/Cas along with the visual BioBrick insert has passed on to the T0 progeny. According to Mendel’s law of segregation, heterozygous dominant transgenic lines and homozygous recessive lines (with no T-DNA and *AtMYB75*) can be easily screened based on the presence and absence, respectively, of purple color. To test the efficiency of this tool kit in tomato, Hu et al. 2019 targeted just 1 site in *SlEIN2*, *SlERFE1* and *SlARF2B* gene, 2 sites in *SlACS2* and *SlACS4* genes and 3 sites in *SlGRAS8* gene. This CRISPR tool kit can easily carry out single, multi-site editing along with multi-gene editing in tomato.

## Classification of CRISPR/Cas system

In archaea and bacteria, the CRISPR/Cas systems render adaptive immunity against foreign nucleic acids. They have a plethora of variable features in various categories like protein composition, pre-crRNA processing and interference, genome locus architecture and mechanisms of adaptation, effector complex structure, etc. The CRISPR/Cas system has been broadly divided into two classes: Class 1 and Class 2, the former being more complex than the later. Class 1 possesses multi-subunit effector complexes and Class 2 with single protein effector modules. Further experimentation and analysis of the Class 2 CRISPR/Cas system led to the discovery of two new types and multiple subtypes. Of the two newly discovered and characterized CRISPR type, the one that solely targeted RNA was the type VI systems. The class 2 systems in some cases display a unique feature where the effector protein is also found responsible in the processing of the pre-crRNA (Koonin et al. 2017).

The primary reason for the variability and fast evolution of the CRISPR/Cas systems is its constant battle with the viruses, which conditions the *cas* genes to evolve fast (Takeuchi et al. 2012), thus leading to a diverse array of gene repertoires and finally to the whole defense infrastructure (Makarova et al. 2011, 2015). More specifically we can conclude that the CRISPR/Cas system has diversified as a response to the competitive coevolution of the anti-CRISPR proteins (Bondy-Denomy et al. 2013, 2015; Pawluk et al. 2016a, b). Comparative sequence analysis, experimental data and structural studies strongly infer that despite being evolutionarily flexible, all CRISPR/Cas variants exhibit common architectural and functional principles and the principle building blocks too exhibit a common ancestry (Makarova et al. 2013). In 2015, a blueprint was proposed which combined the signature genes and the elements of the *Cas* loci. This assigned almost all the CRISPR/Cas loci identified till date, to certain specified subtypes. This updated classification system can be adopted to classify the new varieties of CRISPR/Cas variants discovered from new genomes (Koonin et al. 2017).

### Classes (class 1 and class 2), types and subtypes

It is pretty complicated to classify CRISPR/Cas systems since there is a lack of universal Cas proteins that could have acted as phylogenetic markers. Eventually the classification is based upon multiple factors like signature *cas* genes, layout of the *cas* operons and phylogenies of the conserved Cas proteins (Koonin and Makarova 2019).

The two major classes of the CRISPR/Cas system, namely 1 and 2, have a strong basis of differentiation. The Class 1 group comprises of the multi-subunit crRNA effector complex, whereas the Class 2 group is a collection of the single crRNA effector complex. Diverging further the Class 1 CRISPR/Cas system has been divided into type I, III and IV that is classified into further subtypes. Similarly Class 2 has been divided into types II, V and VI, also subdivided into several subtypes. This classification is based on the mechanism of action of the CRISPR/Cas system, which has been broadly divided into three stages: adaptation, expression and maturation, interference (together form the effector module). The adaptation module performs the spacer acquisition, whereas the effector module performs the pre-crRNA processing, target recognition and cleavage. Both the ‘expression and maturation’ stage and the ‘interference’ stage in type I and type III systems are carried out by a multi-subunit protein complex also known as the Cascade (CRISPR-associated complex for antiviral defense) complex together with the Cas3 nuclease–helicase, the Csm or the Cmr complex for type I, III-A and III-B CRISPR/Cas systems respectively.

In type II, III, V and VI systems, both the expression and maturation stage are performed by a single large polypeptide like the Cas9 in case of type II and Cas10 for type III, whereas for type V systems the process is executed by Cpf1 or other related proteins (Amitai and Sorek 2016). Delving deep within the Class 1 CRISPR/Cas system, type I and type III is predominant in various permutations and combinations in phylogenetically diverse archaea and bacteria (comparatively less frequent), whereas type IV system is seen to be the rare type with a rudimentary CRISPR/Cas loci and does not possess the adaptation module. On the other hand, the distinct type II system of Class 2 is confined to bacteria only (Koonin et al. 2017; Munawar and Ahmad 2021). An interesting fact about CRISPR/Cas systems is that they have been found in viral genomes and plasmids, which suggest horizontal gene transfer (Makarova et al. 2006). Though the Cas proteins are vastly diverse among themselves, all can be grouped into four functional categories: nucleases/recombinases performing spacer acquisition, ribonucleases catalyzing the processing of crRNA guides, a plethora of proteins that bind with the RNA guides resulting in the formation of the crRNP complexes to carry out target surveillance and finally the nucleases that degrade the DNA or RNA targets (Van Der Oost et al. 2014).

We have discussed before that the adaptation module of the CRISPR/Cas system carry out the spacer acquisition from the mobile genetic elements (MGE) and are inserted into the host chromosomal CRISPR array with the assistance of Cas1 and Cas2 (Arslan et al. 2014), although the necessity of these two proteins in type III have not been reported so far. The long pre-crRNA that results from the transcription of the CRISPR array is eventually processed by a cas6 type endoribonuclease, in type I and type III systems into separate crRNAs (Brouns et al. 2008; Carte et al. 2008). On the other side, for crRNA maturation, Cas9 aids the type II systems, along with host RNase III and a tracrRNA (Carte et al. 2014). During the interference stage of type I systems, the Cas proteins couple with the mature crRNAs forming a ribonucleoprotein complex, to degrade the foreign nucleic acid by Cas3 nuclease (Westra et al. 2012), whereas in type II and type III-B systems, this process is carried out by intrinsic nuclease activity of their crRNP complexes (Gasiunas et al. 2012; Jinek et al. 2012). It is interesting to note that the type I and II complexes target DNA while the type III-B complex targets RNA (Staals et al. 2014).

**Fig. 4:**
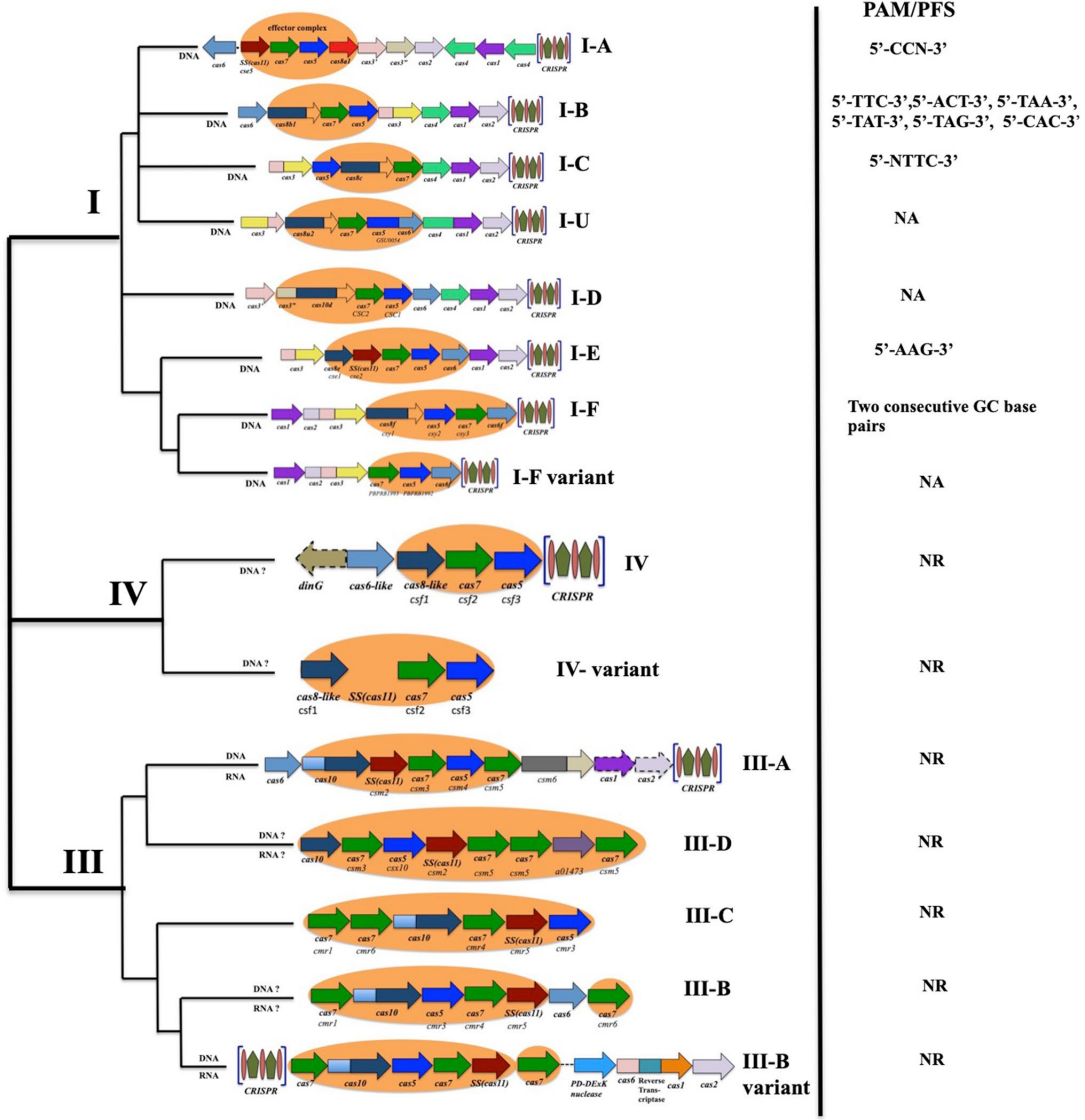
Classification of Class-1 CRISPR/Cas system based on the organization of the CRISPR/Cas loci, domain architectures of the effector proteins and the target (predicted) nucleic acids along with their PAM/PFS sequences; SS—small subunit. The colored arrows represent the corresponding genes and the shaded area represent the Class 1 effector complex. The diversified and common type I, type III are included in the Class I systems found in archaea and less frequently in bacteria. The rare type IV possesses rudimentary CRISPR–Cas loci without the adaptation module. The type I and type III CRISPR–Cas effector complexes possess well-defined architecture, their backbone made of Cas 7 and Cas5, which are paralogous RAMPs (Repeat-Associated Mysterious Proteins, made up of RRM (RNA Recognition Motif) fold with ‘large’ and ‘small’ subunits additionally. Cas6, loosely bound with the effector complex, is an additional RAMP and acts as the repeat-specific RNase in the pre-crRNA processing

Studies have suggested that type I and type III complexes possess some key similarities in their architecture, which points out to the chance of them having a common ancestry. They have a long backbone made of repeat-associated mysterious proteins (RAMPs). These proteins are made up of two parts: RNA recognition motif (RRM) fold plus large and small subunits. The large subunit located at the base of the backbone is Cas8 in case of most type I systems and Cas10 for type III system (Reeks et al. 2013). The backbone consists of multiple copies of Cas7 and a smaller Cas5 like protein. Cas5 couples with 5′ end of the crRNA to interact with the large subunit of both Cas8 (type I) and Cas 10 (type III). The gRNA is housed in the effector complexes that are made up of multiple Cas7 and one Cas5 subunit. It has been reported that the Cas5 subunit binds with the 5′-crRNA and links up to the large subunit of Cas8 in type I and Cas10 for type III. Both type I and type III takes the help of a standalone Cas6 endonuclease in their crRNA processing pathways. The solo Cas6 endonuclease binds to the amorphous pre-crRNA cutting inside each repeat, thus generating intermediate crRNA possessing 5′-3′ repeat derived termini (Charpentier et al. 2015). A comparative study by Khan et al. 2019 (Khan 2019) gives us a clear idea that Cas5/Cas6 is a mandatory element in preprocessing of crRNA, Cas3 and Cascade for further cleavage and crRNA for interference, in case of type I CRISPR/Cas systems. Figure 4 gives a vivid detail of the distinct PAMs required by different subunits of the type I, III, IV systems to carry out target acquisition and recognition. A 5′-CNN-3′ PAM motif is required by type I-A systems (Gudbergsdottir et al. 2011) for interference. Six distinct PAMs are recognized by holoarchaea having type I-B systems. A Cas5-dependent crRNA maturation pathway identified and characterized in type I-C system recognizes an ‘NTTC’ consensus PAM sequence as found in *Bacillus halodurans* (Hyun et al. 2012; Sorek et al. 2013). Type I-E effector complex isolated from *Thermobifida fusca* and *E. coli* (Xiao et al. 2017) binds to a 5′-AAG-3′ PAM sequence. It has been found that type I-E effector complex consists of five Cas proteins, whereas type I-F (found in *Pseudomonas aeruginosa*) consists only four and type I-F targets foreign DNA with the aid of a PAM sequence having two G-C base pairs consecutively (Rollins et al. 2015).

Based on different adaptation, interference and recognition schemes, the type III system has been further divided into four significant subtypes. The type III-A subtype possesses the adaptation-related genes, whereas these are absent in type III-B, C and D systems. Hence, the later three systems incorporate new spacers with total reliance on other systems. Type III shares similarities with type I in pre-crRNA processing strategies and in the structural getup of the crRNP complexes also called the Csm/Cmr complex (Rath et al. 2015). The Csm complex is present in both A and D subtypes while Cmr complex is found in both B and C subtypes. The Cas6 protein plays an integral part in pre-crRNA processing in type III just like type I systems.

**Fig. 5:**
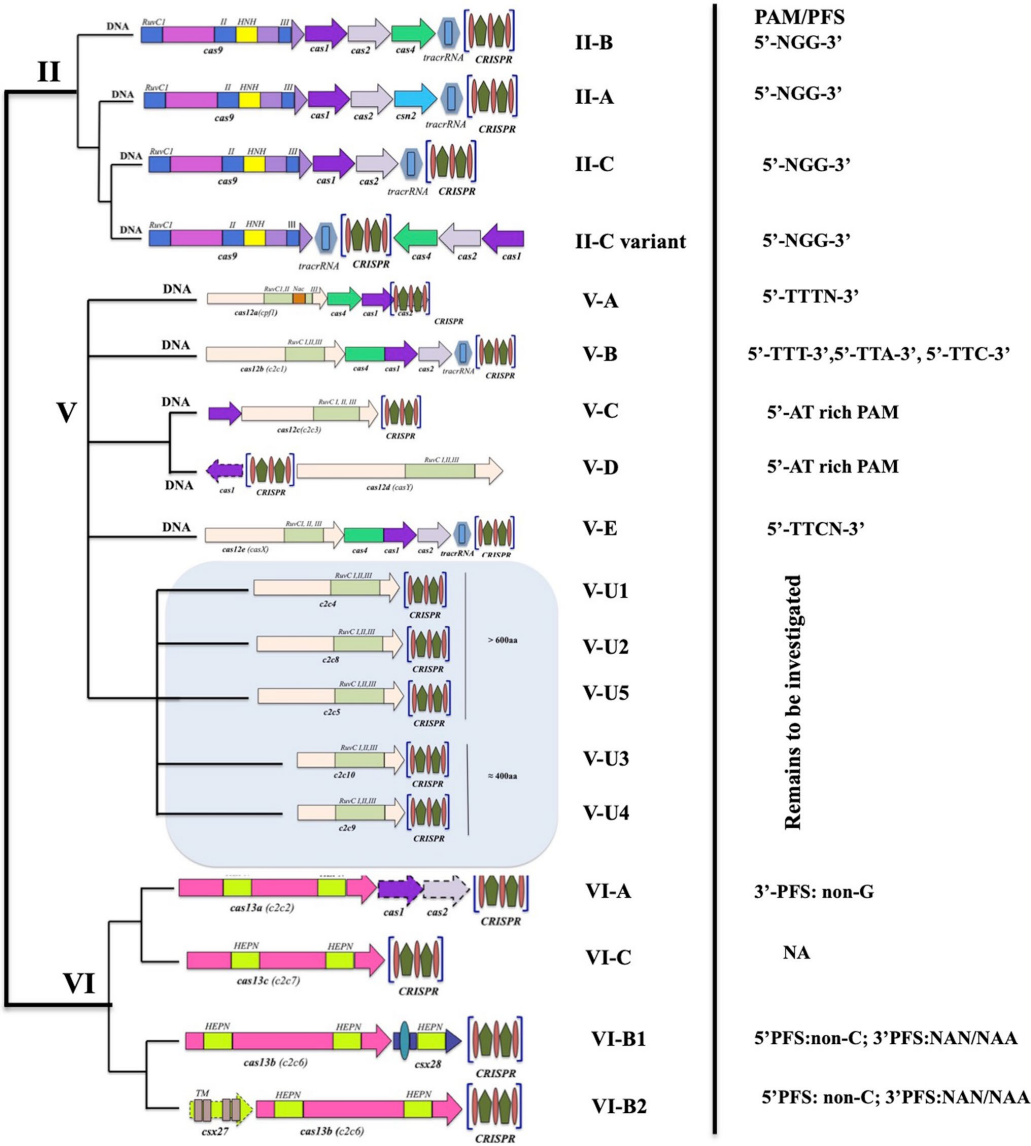
Classification of Class-2 CRISPR/Cas system based on the organization of the CRISPR/Cas loci, domain architectures of the effector proteins and the target (predicted) nucleic acids along with their PAM/PFS sequences; TM-predicted transmembrane segment; HEPN—higher eukaryotes and prokaryotes nucleotide binding. The colored arrows represent the corresponding genes of Class 2 system. The Class 2 effector modules are made up of large, single multi-domain protein making it simpler and better organized than their Class I counterparts

The Class 2 CRISPR/Cas system possesses one large multi-domain protein as the effector complex; hence, it is inferred to be more neatly organized compared to the Class 1 system. The Class 2 system has been further subdivided into three subtypes: type II, type V and type VI (Fig. 5). The well-studied and characterized system in this Class is the type II system possessing the effector Cas9 endonuclease, utilized extensively in genome editing. The entire process of targeting DNA by recruiting Cas9 is orchestrated by crRNA. Yet the trajectory followed by tracrRNA, RNase III and various other factors to carry out the 5′ end processing of crRNA still remains an enigma (Munawar and Ahmad 2021). The most significant feature of type V CRISPR/Cas system is the presence of a single effector protein called Cas12, which has five reported subtypes A to E and a tentative subtype U (Yang et al. 2016). Type V-A CRISPR/Cas system possesses an active endonuclease called the Cas12a (previously known as Cpf1) which can carry out targeted cleavage without the aid of an extra tracrRNA (Zetsche et al. 2015). The ‘TTN’ PAM sequence is the signature sequence for target recognition in case of subtype V-A (Gleditzsch et al. 2019). AT-rich PAM sequence like ‘TTT/TTA/TTC’ is utilized by the type V-B systems, in the line of action to target dsDNA, with the assistance of both tracrRNAs and crRNAs. CasY (Cas12d) and CasX (Cas12e) are the respective characteristic proteins in subtype V-D and V-E and over here CasX needs tracrRNA during interference while CasY needs none. It has also been reported that CasY utilizes a signature ‘TA’ PAM sequence while CasX requires a 5′-TTCN-3′ PAM sequence for target recognition (Burstein et al. 2017). Cas12 effector protein is found to be extremely advantageous in use because of its properties like comparatively smaller size, asymmetric cleavage sites and no requirement of tracrRNA. Hence, Cas12 is a much desired effector protein and experimentation is underway on ways and methods to broaden its horizon of targets by generating variants having different PAM specificities (Gao et al. 2017).

Higher eukaryotes and prokaryotes nucleotide-binding (HEPN) domains are a signature feature in the subtypes of type VI CRISPR/Cas system and it is predicted that they possess RNase activity only. Cas13a (C2c2) was the first protein to be characterized over here. The RNA-targeting activity was well demonstrated for *Leptotrichia shahii* Cas13a (LshCas13a). The presence of Protospacer Flanking Site (PFS) analogous to PAMs for RNA targets was an integral part for carrying out this interference activity (Gleditzsch et al. 2019). A significant difference in proteins among the different subtypes in the type VI system has been noticed. The gradual evolution of type VI-B proteins has been considered to take place from transmembrane proteins since corresponding transmembrane domains have been observed, which makes this subtype unique from type VI proteins (Shmakov et al. 2017; Smargon et al. 2017). Cas13b effector has been identified for *Bergeyella zooohelcum* (BzCas13b) and PFS identification also reported. In a nutshell, it has been inferred that type VI systems are comparatively less stringent than other types in the domain of substrate recognition as it targets RNA solely which has lesser harmful side effects on the cell.

The evolution and diversity observed in the CRISPR/Cas system, a potential arsenal of prokaryotic defense mechanisms against MGE, is the result of the novel effector proteins and novel molecular strategies. The diversity is pronouncedly vivid in the CRISPR/Cas systems of archaea and bacteria. These findings have helped and will help the scientific community for further in vivo and in vitro analyses to fully decipher the mechanisms and strategies on how the intriguing CRISPR/Cas variants function to protect the host cell from lethal MGE invasions.

## Application of CRISPR/Cas9 system

### CRISPR/Cas9 system for resistant breeding and quality improvement in tomato

**Fig. 6:**
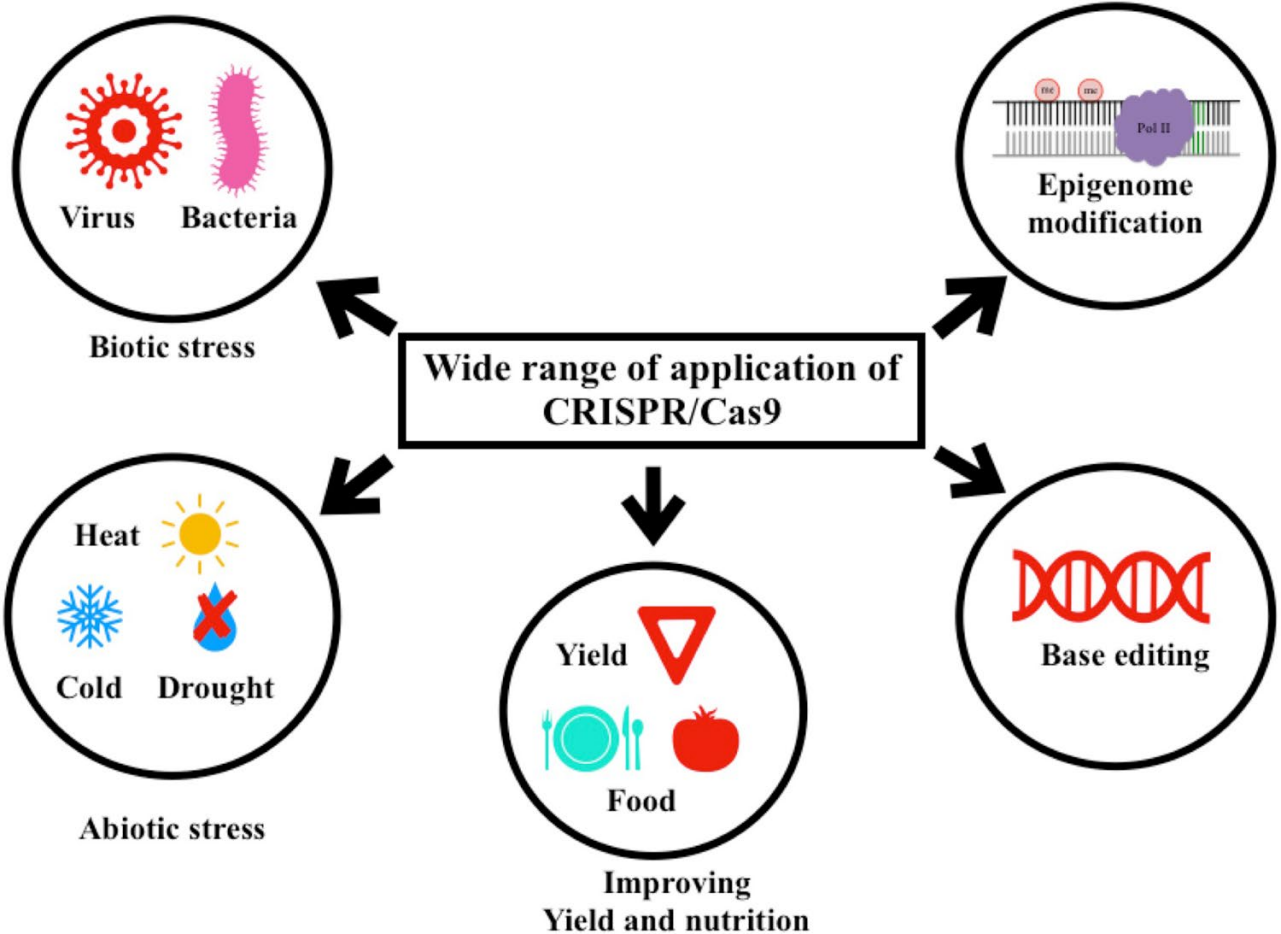
Schematic representation of diverse range of applications of CRISPR/Cas9 technology in tomato breeding

CRISPR/Cas9 has gained much attention in the last decade because of its ease of use and efficiency. It has been used in fleshy fruit model plant ‘tomato’ to enhance several aspects such as yield, nutritional value and tolerance against stress conditions (Fig. 6, Table 2).

**Table 2:** List of genes targeted by CRISPR/Cas9-mediated genome editing in tomato cultivars for enhancing the yield, nutritional values and tolerance to both biotic and abiotic stresses

Tool	Gene	Tomato cultivar	Effect	References
CRISPR	*IAA9*	Micro-Tom, Ailsa Craig	Regenerated mutants exhibited morphological changes in leaf shape and seedless fruit—a characteristic of parthenocarpic tomato	Ueta et al. (2017b)
CRISPR	*PMR4*	Moneymaker	Mutants showed increased resistance against powdery mildew	Santillán Martínez et al. (2020)
CRISPR	*MAPK3*	Ailsa Craig	Susceptibility to gray mold disease	Zhang et al. (2018)
CRISPR	*DCL2b*	Ailsa Craig	Susceptibility to Tomato Mosaic virus (ToMV)	Wang et al. (2018)
CRISPR	*JAZ2*	Moneymaker	Resistance to banana streak virus	Ortigosa et al. (2019)
CRISPR	Homolog of Arabidopsis *SP, CLV3*	M82	Modified inflorescence and plant architectures, Increase in locule number and fruit weight	Rodríguez-Leal et al. (2017)
CRISPR	*SP, WUS*	*S. pimpinellifolium*	Growth habit determination, enlarge fruit size	Li et al. (2018b)
CRISPR	*RIN*	Ailsa craig	Fruit ripening	Ito et al. (2015, 2017, 2020)
CRISPR	*ORRM4*	Micro-Tom	Fruit ripening	Yang et al. (2017)
CRISPR	*AGL6*	Line MP-1 (TYLCV tolerant)	Parthenocarpy	Klap et al. (2017)
CRISPR	*PSY1*	M82, *Yellow flesh e*^*3756*^*, Bicolor*^*cc383*^, *S. pimpinellifolium*^*LA1578*^	Yellow colored fruits	Hayut et al. (2017)
CRISPR	*AGO7*	M82	Change in leaf shape	Brooks et al. (2014)
CRISPR	*BOP1, BOP2, BOP3, TFAM1, TFAM2*	M82	Loss of floral organ abscission, defects in fruit shape, Altered leaf complexity	Xu et al. (2016)
CRISPR	*DELLA*	Micro-Tom	Reduction in serrated leaflets	
	(Shimatani et al. 2017)			
CRISPR	*PDS*	Micro-Tom	Albino phenotype	Pan et al. (2016)
CRISPR	*SHR*	Solanum spp.	Short root	Ron et al. (2014)

*IAA9*, *Aux/IAA9 transcription factor*; *PMR4*, *Powdery Mildew Resistance 4*; *SlMAPK3*, *Solanum lycopersicum MAP kinase3*; *DCL2b, Dicer-like 2b; JAZ2, JASMONATE ZIM DOMAIN2; SP, SELF-PRUNING*; *CLV3, CLAVATA3; WUS, WUSCHEL; RIN, RIPENING INHIBITOR; ORRM4, organelle RNA recognition motif-containing protein4; AGL6, AGAMOUS-LIKE 6; PSY1, PHYTOENE SYNTHASE 1; AGO7, ARGONAUTE 7; BOP1/BOP2/BOP3, BLADE-ON-PETIOLE* family; *TFAM1/TFAM2, mitochondrial transcription factor A; DELLA,* aspartic acid–glutamic acid–leucine–leucine–alanine; *PDS, Phytoene desaturase; SHR, SHORT ROOT*

#### Resistance against abiotic stresses

CRISPR/Cas9 proved to be a valuable tool for identifying previously unknown abiotic stress regulators in tomato plant. The plant hormone brassinosteroid has been known to be associated with various developmental and physiological processes such as cell division, cell elongation, reproduction and seed germination (Divi and Krishna 2009). Yin and colleagues showed that *BRASSINAZOLE-RESISTANT 1* (*BZR1*) is a critical component of brassinosteroid signaling in tomato and it regulates *RESPIRATORY BURST OXIDASE HOMOLOG1* (*RBOH1*) which in turn controls the apoplastic hydrogen peroxide (H_2_O_2_) production and heat stress tolerance (Yin et al. 2018). CRISPR/Cas9-mediated knockout of *BZR1* yields mutant lines with impaired *RBOH1* induction, reduced growth and heat tolerance. In another study, researchers were able to identify the potential role of tomato *NONEXPRESSOR OF PATHOGENESIS-RELATED GENE 1*(*SlNPR1*) in drought stress responses which was previously thought to be involved only in plant’s defense response against pathogens (Li et al. 2019b). CRISPR-*npr1* mutants showed an increased stomatal aperture, higher electrolytic leakage and a reduction in drought tolerance compared to wild-type plants. Wang and her team targeted *MITOGEN-ACTIVATED PROTEIN KINASES* (*SlMAPK3*) in tomato, which revealed its role in drought stress-related responses by transcriptional modulation of other stress-related genes and by protecting cell membranes from oxidative damage (Wang et al. 2017a). CRISPR/Cas9-mediated targeted mutagenesis of *LATERAL ORGAN BOUNDARIES DOMAIN* (*SlLBD40*) resulted in improved drought tolerance in tomato (Liu et al. 2020). Transcriptional activator *C-REPEAT BINDING FACTOR 1* (*CBF1*) has long been associated with cold stress-related regulation of gene expressions (Kanaya et al. 1999; Gilmour et al. 2004), but the detailed mechanism is still not clear in tomato. CRISPR/Cas-mediated knockout of tomato CBF1 (*SlCBF1*) yields mutant lines with severe chilling injuries, higher electrolyte leakage and malondialdehyde levels compared to wild-type plants, contributing to a vivid insight into the molecular mechanism of *SlCBF1*-mediated tomato chilling sensitivity (Rui et al. 2018).

#### Resistance against biotic stresses

Pathogenic microbes such as bacteria, fungus or viruses cause severe damage in crop production. Disease-resistant smart crops can be the only sustainable way to cope with the massive demand in food supply worldwide. Enormous research has been done over the past 25 years to identify the key genes conferring disease resistance in crops. CRISPR/Cas9-mediated inactivation of tomato *DOWNY MILDEW RESISTANCE 6* (*SlDMR6-1*) produced prematurely truncated protein conferring disease resistance against a wide variety of pathogens such as P. syringae, P. capsici and Xanthomonas spp (Zeilmaker et al. 2015; Paula de Toledo Thomazella et al. 2016). In another study, researchers created an improved variety of tomato that can resist powdery mildew fungal pathogen *Oidium neolycopersici.* They took a double guide RNA approach, targeting two regions within the MILDEW-*RESISTANT LOCUS O* (*Mlo*) in tomato for the required loss-of-function mutation (Nekrasov et al. 2017). One of the most devastating diseases in tomato is fusarium wilt disease caused by Fusarium oxysporum f. sp. lycopersici (Sacc.) causing huge losses in tomato production all across the globe. Complementation and knockout strategies using CRISPR/Cas9 revealed a novel tomato gene Solyc08g075770 as the primary reason behind tolerance to fuserium wilt (Prihatna et al. 2018). Another group of researchers demonstrated the potential of CRISPR/CAS9 system to target the coat protein (CP) sequence of *tomato yellow leaf curl virus* (TYLCV) genome in tomato and induce stable and efficient virus interference that remained active across multiple generations (Tashkandi et al. 2018). Methyl jasmonate plays a crucial role in the developmental processes and plant's defense response against various pathogens such as *Botrytis cinerea*. Transcription factor *SlMYC2* is the master regulator of methyl jasmonate signaling pathway (Kazan and Manners 2013). Knockout of *SlMYC2* significantly reduced the expression level of both disease defensive genes and genes related to jasmonic acid pathway, suggesting its prominent role in methyl jasmonate-induced disease resistance in tomato (Shu et al. 2020).

#### Improvement of yield and nutritional quality

Flowering in plants majorly depends upon the day length period, which varies from one season to another. This day length sensitivity limits the geographical range of crop cultivation and yield. CRISPR/Cas9-mediated mutagenesis in *SELF-PRUNING 5G* (*SP5G*) produced the loss of day-length-sensitive tomato lines with rapid flowering and enhanced yield, illustrating the power of this genome editing tool in crop improvement (Soyk et al. 2017). Long shelf life is inarguably the most elite characteristic to tomato breeders for storage and post-harvest produce distribution. Flowering in plants majorly depends upon the day length period, which varies from one season to another (Yu et al. 2017). Parthenocarpy is an industrially important trait in horticultural plants, and the key gene responsible for it is Aux/IAA transcription factor *SlIAA9* (Wang et al. 2005). CRISPR/Cas9-mediated targeted mutagenesis in *SlIAA9* produced parthenocarpic tomato lines and the trait is segregated into the next generation successfully (Ueta et al. 2017a). Wild crop varieties have been domesticated for decades to cope with the growing population and demand in the food supply, which leads to loss of genetic diversity and resistance against biotic and abiotic stresses. Researchers were able to domesticate a wild variety of tomato (*Solanum pimpinellifolium*) with improved size, number and nutritional value by targeting six important loci *(SELF-PRUNING, OVATE, FASCIATED, FRUIT WEIGHT, MULTIFLORA, LYCOPENE BETA CYCLASE)* using CRISPR/Cas9 (Zsögön et al. 2018). GABA (γ-aminobutyric acid) homeostasis is crucial for plants' developmental processes and is regulated via a GABA shunt (Takayama and Ezura 2015). Five key genes regulating this shunt were targeted using a multiplex pYLCRISPR/Cas9 system yielding mutant tomato lines with a significant increase in GABA accumulation in both leaves and fruits (Li et al. 2018a).

### CRISPR/Cas9 system for targeting polyploid crops

CRISPR/Cas9 is the most widely utilized genome editing tool in plants, but its editing efficiency has varied widely and dramatically. The editing in polyploid crops is especially challenging because the paralogs and orthologs present there, having functional redundancy, require a cumbersome simultaneous knockout of all copies of genes, which are functionally similar. Two crucial factors that directly affect the mutagenic frequency of polyploid crops are: optimization of Cas9 codon, promoters and target sequence composition (Zhang et al. 2014; Ma et al. 2015, 2016; Yan et al. 2015; Mao et al. 2016). The most difficult task in this procedure is designing the sgRNAs, which is extremely challenging in case of polyploids than in diploids. Though a couple of sgRNA designing tools are prevalent in the market (CRISPR-P, CRISPR-P_2.0_), they cannot be used smoothly in polyploidy crops (Lei et al. 2014; Liu et al. 2017). The strategy best adopted for the simultaneous knockout of orthologs and paralogs would be to design sgRNAs picked up from a conserved domain, which targets the entire collection of gene copies. Manual designing of sgRNAs targeting one particular gene copy or all copies can be done after performing detailed sequence analysis. It happens sometimes that a large cross section of homologous genes do not possess a conserved site, those genes are divided into several groups and sgRNAs are designed on the basis of the conserved site of each group (Zaman et al. 2019). Apart from targeted mutagenesis, another noteworthy application of CRISPR/Cas-mediated genome editing for improvement of polyploid crops is the targeted substitution of unwanted alleles with the desired ones (Schaart et al. 2021).

CRISPR-Cpf1, a new class of CRISPR system, is quite analogous to Cas9 and enables the editing of AT-rich regions like the 5′ and 3′ UTRs and promoter domains. The fundamental advantage of Cpf1 over Cas9 is that the Cpf1 crRNA is shorter than the spCas9 sgRNA by 60 nucleotides, and at the same time, no tracrRNA is needed (Fonfara et al. 2016), hence facilitating multiplex gene editing (Wang et al. 2017b). Additional tracrRNA is not required by Cpf1 to form a mature crRNA. Unlike Cas9 that recognizes G-rich PAM sequences, Cpf1 recognizes T-rich PAM sequences. Finally, it was also observed that Cas9 endonuclease generates blunt ends, whereas Cpf1 endonuclease produces cohesive ends (Xu et al. 2019). Researchers have recently identified numerous expanded PAM of Cas9 and Cpf1 variants, which will eventually facilitate sgRNA designing for the genome editing of polyploid crops. Another noteworthy application of crop improvement is CRISPR/Cas-mediated precise base editing. An addition of a ‘base editing’ function to CRISPR/Cas9 can induce a C→T and G→A conversion. The base editing system of wheat can be superimposed to carry out site-specific modification in other polyploid crops.

Rodríguez-Leal et al. 2017 took a significant step ahead by combining the diverse behaviors of CRISPR/Cas9 to engineer quantitative trait loci variation (QTL) by performing mutations in the *cis*-regulatory regions. The huge collection of *cis*-regulatory alleles that was created revealed that a humongous number of quantitative variations could be achieved by remodeling the expression of individual genes. Thus, performing different permutations in promoters of various developmental regulators can modify diverse traits of crop plants. *SlCLV3* promoter alleles were characterized, which consequently provided fundamental insights on numerous angles like the complex structure of the *cis*-regulatory regions, regulation of transcription and finally the control of quantitative traits. A study was performed, where a bidirectional strategy carried out (Li et al. 2018c) which involved promoting the lycopene content of tomato on the one hand and blocking the conversion of lycopene to β- and α-carotene. Five candidate genes regulating the carotenoid metabolic pathway were edited using the CRISPR/Cas9 system followed by *Agrobacterium tumefaciens*-mediated transformation. The breakthrough result obtained there was a 5.1-fold increase in the lycopene content of tomato. The homozygous trait was transmitted successfully to the succeeding generations. Breakthrough research by (Liang et al. 2017) on bread wheat makes it clear that it is more than possible to utilize CRISPR/Cas ribonucleoproteins (RNPs) for selection-free site-directed mutagenesis by embryo bombardment. To stop all possibilities of transgene integration and to minimize off-target mutations are the two essential points of optimization for making CRISPR/Cas9 system a highly precise method for crop breeding. This technique was a breakthrough since it involved the delivery of active Cas9-gRNA complexes embedded on gold particles into maize cells, along with the recovery of mutant plants without selection, makes the approach extremely user friendly for genome editing in major crops.

### CRISPR/Cas9 for epigenome modification and base editing

Epigenetics refers to the heritable changes in the gene expression level, generating phenotypic variation without altering the nucleotides in the DNA sequence. Three mechanisms tightly control epigenetic regulation: DNA methylation, post-translational histone modification and the action of non-coding RNAs (short-interfering RNAs, siRNAs; microRNAs, miRNAs), which can lead to changes in chromatin structure without affecting the DNA sequence itself. This sort of gene regulation plays a crucial role in plant’s developmental stages and its response toward biotic and abiotic stresses (Fujimoto et al. 2012). DNA methylation in plants involves the addition of a methyl group on the 5′carbon of cytosine base to form 5′methylcytosine and it can happen in both symmetric CG, CHG and asymmetric CHH context (H = A/C/T; A, adenine; G, guanine; C, cytosine; T, thymine) (Feng et al. 2010). DNA methylation within the coding sequence leads to altered gene expressions, whereas methylation in the promoter region results in gene silencing. A dynamic interplay between DNA methylation and demethylation is very crucial for silencing deleterious transposon insertions and regulating overall gene expressions during the developmental stages of plants (Van Oosten et al. 2014).

**Fig. 7:**
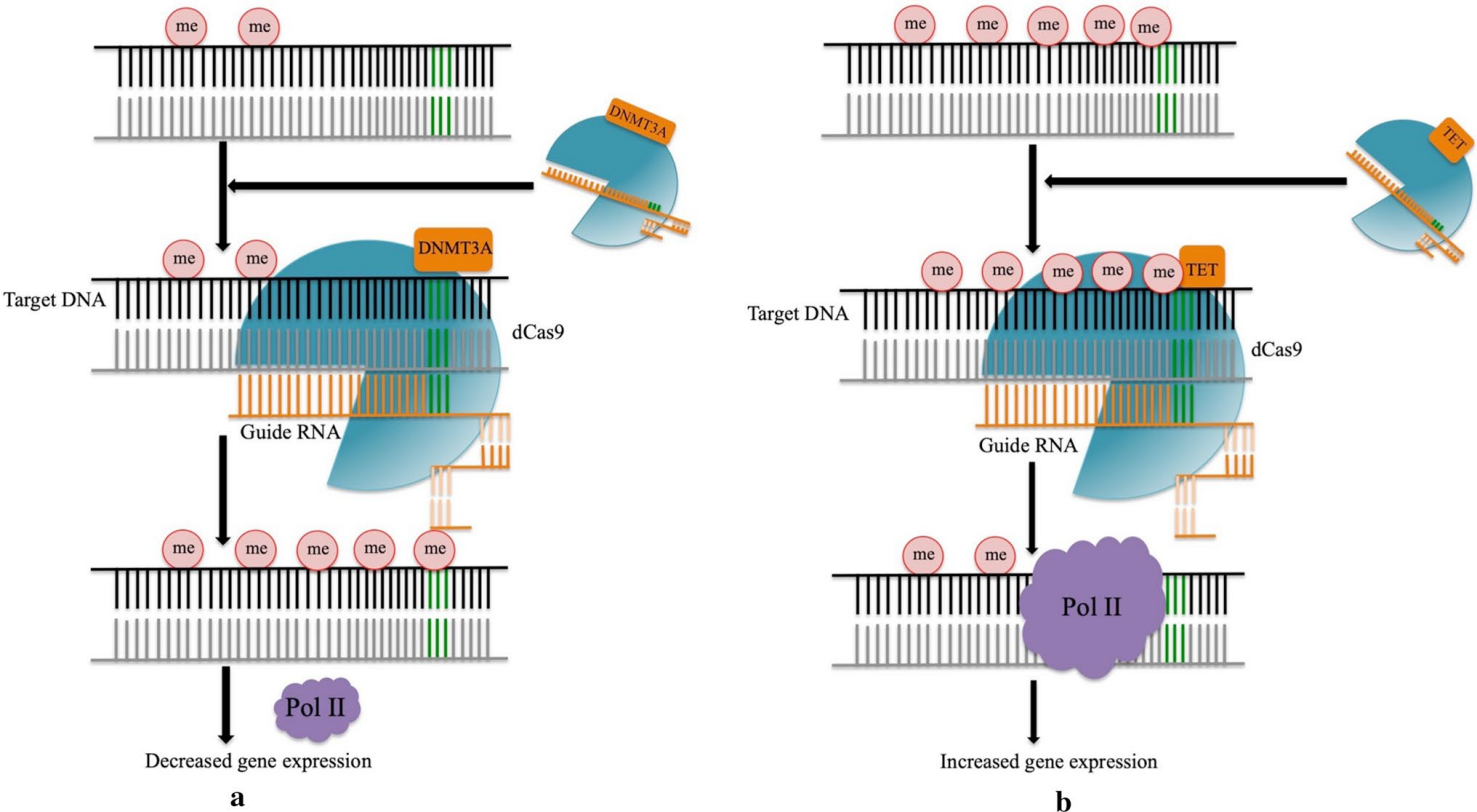
dCas9-mediated methylation/demethylation process. dCas9 is the nuclease inactivated Cas9 variant that basically serves as DNA-binding protein. DNMT3A (DNA methyltransferase) and TET1 (ten-eleven translocation dioxygenase 1) are the epieffectors for DNA methylation and demethylation, respectively. Specific guide RNA coupled with dCas9-epieffector complex is able to methylate/demethylate the targeted dsDNA. **a** dCas9-DNMT3A-mediated methylation of dsDNA resulting in decreased level of gene expression. **b** dCas9-TET1-mediated demethylation of dsDNA resulting in increased level of gene expression

Since its inception, Cas9 has been very rapidly and widely accepted as a tool for genome editing. Initially dCas9-the nuclease inactivated Cas9 variant carrying point mutations in the HNH/RuvC-like catalytic domain was created to elucidate their roles in dsDNA cleavage (Jinek et al. 2012). But later on, it was adopted as a DNA-binding platform for a diverse range of functions such as epigenetic modifications (Hilton et al. 2015; Kearns et al. 2015) and gene-expression modulations (Cheng et al. 2013). The strategy to use CRISPR/Cas in epigenetic modification is to fuse the dCas9 with a transcriptional activator or repressor domain known to have epigenetic effects (epieffector). Specifically designed gRNA coupled with dCas9-epieffector complex can achieve the required methylation/demethylation at the DNA level for the desired change in phenotype or trait (Fig. 7a, b). The conventional CRISPR/Cas system has been successfully applied to a wide range of crops for improving yield, nutritional value, and stress and disease resistance. However, one of the major concerns regarding this technology is the risk of off targeting, resulting in unpredictable mutations. The conventional CRISPR/Cas system has been successfully applied to a wide range of crops for improving yield, nutritional value, and stress and disease resistance (Mlambo et al. 2018). Epigenetic modulation leads to genetic gain of function, which could speed up the process of domestication of wild species with improved yield, nutrition, fruit and seed numbers (Springer 2013). Induced methylation/demethylation could impact hybrid breeding and induce new gene expression patterns in next-generation offspring, thereby controlling their phenotypes (Stroud et al. 2013; Stelpflug et al. 2014). Papikian et al. 2019 successfully triggered early flowering phenotype in *Arabidopsis thaliana* using dCas9-SunTag system-mediated induction of FWA promoter. dCas9-mediated targeted induction of DNA methylation to alter gene expression level of those genes that negatively affects the desired traits and yield in crops could be a potential way to achieve food security in future.

Base editing refers to a novel CRISPR/Cas-mediated genome editing technique to mutate a single base without the need for double-stranded breaks (DSBs) or homology directed repair. With the aid of CRISPR/Cas base editors all four transition mutations (A to G, T to C, G to A and C to T) can be achieved. Conventional CRISPR/Cas-mediated indels by generating DSBs has greater risk of off-targeting, while fusing it with base editors to dCas9 or nCas9 (D10A nickase), desired point nutation can be achieved minimizing the risk of off-targeting (Komor et al. 2017). For instance, fusing cytidine deaminase which operates only on single-stranded DNA, to a nuclease inactivated dCas9 can achieve C to U conversion with great precision (Jiang et al. 2016; Komor et al. 2016). dCas9/nCas9-mediated adenine or cytosine base editing has been successfully applied to many plant species including tomato, rice, wheat, maize, brassica over last few years, proving it to be an alternative tool for crop improvement beside the conventional CRISPR/Cas9 (Shimatani et al. 2017; Zong et al. 2017; Hua et al. 2018; Kang et al. 2018).

## Conclusion

As per ‘Food and Agricultural Organization’ (FAO), the world population is increasing in an exponential manner and it is estimated that it will reach approx. 9.1 billion by 2050 (United Nations World Population Prospects: FAO, 2019). The food production capacity needs to be increased by at least 70% to feed this huge population. Conventional breeding methods for agronomic crops such as tomato would not be able to keep up with the pace and improved variety of disease-resistant smart crops will be a necessity to address the food security of the world. In this context, next-generation genome editing technology like CRISPR/Cas gains importance, which has been used in tomato for enhancing the yield, nutritional values and tolerance to both biotic and abiotic stresses.

In recent years, researchers around the globe have been able to identify the presence of two different classes of CRISPR/Cas systems in bacteria and archaea. The diversity leading to the classification of CRISPR/Cas systems is the result of novel effector proteins, locus architecture and unique molecular mechanisms. The most mention worthy mechanisms being ‘the sole RNA targeting’ seen in the type VI systems establishing a direct link between CRISPR immunity and dormancy induction, the pre-crRNA processing by the type V-A, VI-A effector proteins and the activity of CRISPR-associated reverse transcriptase in type III systems carried out for RNA adaptation (Sukrit et al. 2016). The discovery of Casposons and type V-U loci opens doors for further investigation of the pathway of gradual evolution of the CRISPR/Cas system which finally leads to the formation of adaptive immunity from mobile genetic elements. The past two decades have witnessed an immense development in CRISPR/Cas research including PAM-free CRISPR/Cas system, dCas9-mediated epigenome modification and targeted base editing, yet several key questions remain to be answered like the missing link connecting interference and adaptation in primed spacer acquisition, the mystery behind the horizontal transfer of CRISPR and many more. A lot of breakthrough research is going on globally to elucidate numerous arcane and outstanding questions of this field. It appears that CRISPR/Cas system will not be barricaded by genomic complexity, GM controversy, government sanctions, etc. and is here to stay.

## Abbreviations

RNAi: RNA interference
ZFN: Zinc finger nuclease
TALEN: Transcription activator-like effector nuclease
CRISPR: Clustered regularly interspaced short palindromic repeats
GM: Genetically modified
GMO: Genetically modified organism
RNP: Ribonucleoprotein
NHEJ: Non-homologous end joining
PAM: Protospacer adjacent motif
DBD: DNA binding domains
DSB: Double-stranded break
CRISPRi: CRISPR interference
crRNA: CRISPR RNA
tracrRNA: Transactivating CRISPR RNA
gRNA: Guide RNA
sgRNA: Single guide RNA
MGE: Mobile genetic element
RRM: RNA recognition motif
RAMP: Repeat-associated mysterious proteins
HEPN: Higher eukaryotes and prokaryotes nucleotide-binding domain
PFS: Protospacer Flanking Site
CP: Coat protein
TYLCV: Tomato yellow leaf curl virus
QTL: Quantitative trait loci

## References

[CR1] Amitai G, Sorek R (2016). CRISPR-Cas adaptation: insights into the mechanism of action. Nat Rev Microbiol.

[CR2] Arslan Z, Hermanns V, Wurm R (2014). Detection and characterization of spacer integration intermediates in type I-E CRISPR-Cas system. Nucleic Acids Res.

[CR3] Barrangou R, Fremaux C, Deveau H, et al (2007) CRISPR provides acquired resistance against viruses in prokaryotes. Science 315(80):1709–1712. 10.1126/science.1138140 10.1126/science.1138140 17379808

[CR4] Boettcher M, McManus MT (2015). Choosing the right tool for the job: RNAi, TALEN, or CRISPR. Mol Cell.

[CR5] Bolotin A, Quinquis B, Sorokin A, Ehrlich SD (2005) Clustered regularly interspaced short palindrome repeats ( CRISPRs ) have spacers of extrachromosomal origin. 1066:2551–2561. 10.1099/mic.0.28048-0 10.1099/mic.0.28048-0 16079334

[CR6] Bondy-Denomy J, Pawluk A, Maxwell KL, Davidson AR (2013). Bacteriophage genes that inactivate the CRISPR/Cas bacterial immune system. Nature.

[CR7] Bondy-Denomy J, Garcia B, Strum S (2015). Multiple mechanisms for CRISPR-Cas inhibition by anti-CRISPR proteins. Nature.

[CR8] Bonny S (2003). Why are most Europeans opposed to GMOs? Factors explaining rejection in France and Europe. Electron J Biotechnol.

[CR9] Brooks C, Nekrasov V, Lipppman ZB, Van Eck J (2014). Efficient gene editing in tomato in the first generation using the clustered regularly interspaced short palindromic repeats/CRISPR-associated9 system. Plant Physiol.

[CR10] Brouns SJJ, Jore M, Lundgren M, et al (2008) Small CRISPR RNAs guide antiviral defense in prokaryotes. Science (80) 960–963 10.1126/science.1159689 PMC5898235 18703739

[CR11] Burmistrz M, Krakowski K, Krawczyk-Balska A (2020) RNA-targeting CRISPR—Cas Systems And Their Applications 10.3390/ijms21031122 PMC7036953 32046217

[CR12] Burstein D, Harrington LB, Strutt SC (2017). New CRISPR-Cas systems from uncultivated microbes. Nature.

[CR13] Carte J, Wang R, Li H (2008). Cas6 is an endoribonuclease that generates guide RNAs for invader defense in prokaryotes. Genes Dev.

[CR14] Carte J, Christopher RT, Smith JT (2014). NIH public access. Mol Microbiol.

[CR15] Čermák T, Curtin SJ, Gil-Humanes J (2017). A multipurpose toolkit to enable advanced genome engineering in plants. Plant Cell.

[CR16] Čermák T, Baltes NJ, Čegan R, et al (2015) High-frequency, precise modification of the tomato genome. Genome Biol 16. 10.1186/s13059-015-0796-9 10.1186/s13059-015-0796-9 PMC4635538 26541286

[CR17] Chakrabarti AM, Henser-Brownhill T, Monserrat J (2019). Target-Specific precision of CRISPR-mediated genome editing. Mol Cell.

[CR18] Charpentier E, Richter H, van der Oost J, White MF (2015). Biogenesis pathways of RNA guides in archaeal and bacterial CRISPR-Cas adaptive immunity. FEMS Microbiol Rev.

[CR19] Chatterjee P, Jakimo N, Lee J (2020). An engineered ScCas9 with broad PAM range and high specificity and activity. Nat Biotechnol.

[CR20] Chen S, Sanjana NE, Zheng K (2015). Genome-wide CRISPR screen in a mouse model of tumor growth and metastasis. Cell.

[CR21] Cheng AW, Wang H, Yang H (2013). Multiplexed activation of endogenous genes by CRISPR-on, an RNA-guided transcriptional activator system. Cell Res.

[CR22] Collias D, Beisel CL (2021). CRISPR technologies and the search for the PAM-free nuclease. Nat Commun.

[CR23] Council EASA (2013) Planting the future : opportunities and challenges for using crop genetic improvement technologies for sustainable agriculture

[CR24] Deltcheva E, Chylinski K, Sharma CM (2011). CRISPR RNA maturation by trans-encoded small RNA and host factor RNase III. Nature.

[CR25] Divi UK, Krishna P (2009). Brassinosteroid: a biotechnological target for enhancing crop yield and stress tolerance. N Biotechnol.

[CR26] Doudna JA, Charpentier E (2014) The new frontier of genome engineering with CRISPR-Cas9. Science (80):346. 10.1126/science.1258096 10.1126/science.1258096 25430774

[CR27] Engler C, Kandzia R, Marillonnet S (2008) A one pot, one step, precision cloning method with high throughput capability. PLoS One 3. 10.1371/journal.pone.0003647 10.1371/journal.pone.0003647 PMC2574415 18985154

[CR28] Feng S, Jacobsen SE, Reik W (2010) Epigenetic reprogramming in plant and animal development. Science (80- ) 330:622–627. 10.1126/science.1190614 10.1126/science.1190614 PMC2989926 21030646

[CR29] Fonfara I, Richter H, BratoviÄ M (2016). The CRISPR-associated DNA-cleaving enzyme Cpf1 also processes precursor CRISPR RNA. Nature.

[CR30] Fujimoto R, Sasaki T, Ishikawa R (2012). Molecular mechanisms of epigenetic variation in plants. Int J Mol Sci.

[CR31] Gao L, Cox DBT, Yan WX (2017). Engineered Cpf1 variants with altered PAM specificities increase genome targeting range. Nat Biotechnol.

[CR32] Gasiunas G, Barrangou R, Horvath P, Siksnys V (2012). Cas9-crRNA ribonucleoprotein complex mediates specific DNA cleavage for adaptive immunity in bacteria. Proc Natl Acad Sci U S A.

[CR33] Gilbert LA, Horlbeck MA, Adamson B et al (2015) Genome-scale CRISPR-mediated control of gene repression and activation 159:647–661. 10.1016/j.cell.2014.09.029.Genome-Scale 10.1016/j.cell.2014.09.029 PMC4253859 25307932

[CR34] Gilmour SJ, Fowler SG, Thomashow MF (2004). Arabidopsis transcriptional activators CBF1, CBF2, and CBF3 have matching functional activities. Plant Mol Biol.

[CR35] Gleditzsch D, Pausch P, Müller-Esparza H (2019). PAM identification by CRISPR-Cas effector complexes: diversified mechanisms and structures. RNA Biol.

[CR36] Globus R, Qimron U (2018). A technological and regulatory outlook on CRISPR crop editing. J Cell Biochem.

[CR37] Gudbergsdottir S, Deng L, Chen Z (2011). Dynamic properties of the Sulfolobus CRISPR/Cas and CRISPR/Cmr systems when challenged with vector-borne viral and plasmid genes and protospacers. Mol Microbiol.

[CR38] Hartwell LH, Goldberg ML, Fischer JA, Hood L (2018) Genetics from genes to genomes, 6th edn

[CR39] Hayut SF, Bessudo CM, Levy AA (2017). Targeted recombination between homologous chromosomes for precise breeding in tomato. Nat Commun.

[CR40] Heigwer F, Port F, Boutros M (2018). Rna interference (RNAi) screening in Drosophila. Genetics.

[CR41] Hilton IB, D’Ippolito AM, Vockley CM (2015). Epigenome editing by a CRISPR-Cas9-based acetyltransferase activates genes from promoters and enhancers. Nat Biotechnol.

[CR42] Hu N, Xian Z, Li N et al (2019) Rapid and user-friendly open-source CRISPR/Cas9 system for single- or multi-site editing of tomato genome. Hortic Res 6. 10.1038/s41438-018-0082-6 10.1038/s41438-018-0082-6 PMC6312546 30603093

[CR43] Hua K, Tao X, Yuan F (2018). Precise A·T to G·C base editing in the rice genome. Mol Plant.

[CR44] Huang S, Weigel D, Beachy RN, Li J (2016). A proposed regulatory framework for genome-edited crops. Nat Genet.

[CR45] Huibers RP, Loonen AEHM, Gao D (2013). Powdery mildew resistance in tomato by impairment of SlPMR4 and SlDMR1. PLoS ONE.

[CR46] Hyun NK, Haitjema C, Xueqi L (2012). NIH public access. Structure.

[CR47] Ishino Y, Shinagawa H, Makino K (1987). Nucleotide sequence of the iap gene, responsible for alkaline phosphatase isoenzyme conversion in Escherichia coli, and identification of the gene product. J Bacteriol.

[CR48] Ito Y, Nishizawa-Yokoi A, Endo M (2015). CRISPR/Cas9-mediated mutagenesis of the RIN locus that regulates tomato fruit ripening. Biochem Biophys Res Commun.

[CR49] Ito Y, Nishizawa-Yokoi A, Endo M (2017). Re-evaluation of the rin mutation and the role of RIN in the induction of tomato ripening. Nat Plants.

[CR50] Ito Y, Sekiyama Y, Nakayama H (2020). Allelic mutations in the ripening-inhibitor locus generate extensive variation in tomato ripening. Plant Physiol.

[CR51] Jiang F, Taylor DW, Chen JS et al (2016) Structures of a CRISPR-Cas9 R-loop complex primed for DNA cleavage. Science 351(80):867–871. 10.1126/science.aad8282 10.1126/science.aad8282 PMC5111852 26841432

[CR52] Jinek M, Chylinski K, Fonfara I et al (2012) A programmable dual-RNA—guided DNA endonuclease in adaptive bacterial immunity. 337:816–822 10.1126/science.1225829 PMC6286148 22745249

[CR53] Kanaya E, Nakajima N, Morikawa K (1999). Characterization of the transcriptional activator CBF1 from Arabidopsis thaliana: evidence for cold denaturation in regions outside of the DNA binding domain. J Biol Chem.

[CR54] Kang BC, Yun JY, Kim ST (2018). Precision genome engineering through adenine base editing in plants. Nat Plants.

[CR55] Kazan K, Manners JM (2013). MYC2: the master in action. Mol Plant.

[CR56] Kearns NA, Pham H, Tabak B (2015). Functional annotation of native enhancers with a Cas9-histone demethylase fusion. Nat Methods.

[CR57] Khan SH (2019). Genome-editing technologies: concept, pros, and cons of various genome-editing techniques and bioethical concerns for clinical application. Mol Ther Nucleic Acids.

[CR58] Klap C, Yeshayahou E, Bolger AM (2017). Tomato facultative parthenocarpy results from SlAGAMOUS-LIKE 6 loss of function. Plant Biotechnol J.

[CR59] Komor AC, Kim YB, Packer MS (2016). Programmable editing of a target base in genomic DNA without double-stranded DNA cleavage. Nature.

[CR60] Komor AC, Zhao KT, Packer MS (2017). Improved base excision repair inhibition and bacteriophage Mu Gam protein yields C:G-to-T: a base editors with higher efficiency and product purity. Sci Adv.

[CR61] Koonin EV, Makarova KS, Zhang F (2017). Diversity, classification and evolution of CRISPR-Cas systems. Curr Opin Microbiol.

[CR62] Koonin EV, Makarova KS (2019) Origins and evolution of CRISPR-Cas systems. Philos Trans B 374. 10.32416/article_5cd16d07bcc677.54712794 10.1098/rstb.2018.0087 PMC6452270 30905284

[CR63] Lei Y, Lu L, Liu HY (2014). CRISPR-P: a web tool for synthetic single-guide RNA design of CRISPR-system in plants. Mol Plant.

[CR64] Li Y, Qin L, Zhao J (2017). SlMAPK3 enhances tolerance to tomato yellow leaf curl virus (TYLCV) by regulating salicylic acid and jasmonic acid signaling in tomato (Solanum lycopersicum). PLoS ONE.

[CR65] Li R, Li R, Li X (2018). Multiplexed CRISPR/Cas9-mediated metabolic engineering of γ-aminobutyric acid levels in Solanum lycopersicum. Plant Biotechnol J.

[CR66] Li X, Wang Y, Chen S (2018). Lycopene is enriched in tomato fruit by CRISPR/Cas9-mediated multiplex genome editing. Front Plant Sci.

[CR67] Li G, Liu YG, Chen Y (2019). Genome-editing technologies: the gap between application and policy. Sci China Life Sci.

[CR68] Li R, Liu C, Zhao R (2019). CRISPR/Cas9-Mediated SlNPR1 mutagenesis reduces tomato plant drought tolerance. BMC Plant Biol.

[CR69] Li T, Yang X, Yu Y et al (2018b) Domestication of wild tomato is accelerated by genome editing 36. 10.1038/nbt.4273 10.1038/nbt.4273 30272676

[CR70] Liang Z, Chen K, Li T (2017). Efficient DNA-free genome editing of bread wheat using CRISPR/Cas9 ribonucleoprotein complexes. Nat Commun.

[CR71] Liu H, Ding Y, Zhou Y (2017). CRISPR-P 2.0: An improved CRISPR-Cas9 tool for genome editing in plants. Mol Plant.

[CR72] Liu L, Zhang J, Xu J (2020). CRISPR/Cas9 targeted mutagenesis of SlLBD40, a lateral organ boundaries domain transcription factor, enhances drought tolerance in tomato. Plant Sci.

[CR73] Lozano-Juste J, Cutler SR (2014). Plant genome engineering in full bloom. Trends Plant Sci.

[CR74] Ma X, Zhang Q, Zhu Q (2015). A robust CRISPR/Cas9 system for convenient, high-efficiency multiplex genome editing in monocot and dicot plants. Mol Plant.

[CR75] Ma X, Zhu Q, Chen Y, Liu YG (2016). CRISPR/Cas9 platforms for genome editing in plants: developments and applications. Mol Plant.

[CR76] Makarova KS, Haft DH, Barrangou R (2011). Evolution and classification of the CRISPR-Cas systems. Nat Rev Microbiol.

[CR77] Makarova KS, Wolf YI, Koonin EV (2013). systems. Biochem Soc Trans.

[CR78] Makarova KS, Wolf YI, Alkhnbashi OS (2015). An updated evolutionary classification of CRISPR-Cas systems. Nat Rev Microbiol.

[CR79] Makarova KS, Grishin N V, Shabalina SA et al (2006) A putative RNA-interference-based immune system in prokaryotes : computational analysis of the predicted enzymatic machinery , functional analogies with eukaryotic RNAi , and hypothetical mechanisms of action 26:1–26. 10.1186/1745-6150-1-7 10.1186/1745-6150-1-7 PMC1462988 16545108

[CR80] Mao Y, Zhang Z, Feng Z (2016). Development of germ-line-specific CRISPR-Cas9 systems to improve the production of heritable gene modifications in Arabidopsis. Plant Biotechnol J.

[CR81] Mao Y, Botella JR, Liu Y, Zhu JK (2019). Gene editing in plants: progress and challenges. Natl Sci Rev.

[CR82] Mianné J, Chessum L, Kumar S (2016). Correction of the auditory phenotype in C57BL/6N mice via CRISPR/Cas9-mediated homology directed repair. Genome Med.

[CR83] Mlambo T, Nitsch S, Hildenbeutel M (2018). Designer epigenome modifiers enable robust and sustained gene silencing in clinically relevant human cells. Nucleic Acids Res.

[CR84] Mojica FJM, Díez-Villaseñor C, Soria E, Juez G (2000). Biological significance of a family of regularly spaced repeats in the genomes of Archaea, Bacteria and mitochondria. Mol Microbiol.

[CR85] Munawar N, Ahmad A (2021) CRISPR/Cas system: an introduction. Springer Nature Singapore Pte Ltd

[CR86] Nekrasov V, Wang C, Win J (2017). Rapid generation of a transgene-free powdery mildew resistant tomato by genome deletion. Sci Rep.

[CR87] Orack JC, Deleidi M, Pitt D (2015). Concise review: modeling multiple sclerosis with stem cell biological platforms: toward functional validation of cellular and molecular phenotypes in inflammation-induced neurodegeneration. Stem Cells Transl Med.

[CR88] Ortigosa A, Gimenez-Ibanez S, Leonhardt N, Solano R (2019). Design of a bacterial speck resistant tomato by CRISPR/Cas9-mediated editing of SlJAZ2. Plant Biotechnol J.

[CR89] Pan C, Ye L, Qin L et al (2016) CRISPR / Cas9-mediated efficient and heritable targeted mutagenesis in tomato plants in the first and later generations. 1–10. 10.1038/srep24765 10.1038/srep24765 PMC4838866 27097775

[CR90] Papikian A, Liu W, Gallego-Bartolomé J, Jacobsen SE (2019). Site-specific manipulation of Arabidopsis loci using CRISPR-Cas9 SunTag systems. Nat Commun.

[CR91] Pawluk A, Amrani N, Zhang Y (2016). Naturally occurring off-switches for CRISPR-Cas9. Cell.

[CR92] Pawluk A, Staals RHJ, Taylor C (2016). Inactivation of CRISPR-Cas systems by anti-CRISPR proteins in diverse bacterial species. Nat Microbiol.

[CR93] Pourcel C, Salvignol G, Vergnaud G (2005) CRISPR elements in Yersinia pestis acquire new repeats by preferential uptake of bacteriophage DNA , and provide additional tools for evolutionary studies 653–663. 10.1099/mic.0.27437-0 10.1099/mic.0.27437-0 15758212

[CR94] Prihatna C, Barbetti MJ, Barker SJ (2018). A novel tomato Fusarium wilt tolerance gene. Front Microbiol.

[CR95] Rath D, Amlinger L, Rath A, Lundgren M (2015). The CRISPR-Cas immune system: biology, mechanisms and applications. Biochimie.

[CR96] Reeks J, Naismith JH, White MF (2013). CRISPR interference: a structural perspective. Biochem J.

[CR97] Rodríguez-Leal D, Lemmon ZH, Man J (2017). Engineering quantitative trait variation for crop improvement by genome editing. Cell.

[CR98] Rollins MF, Schuman JT, Paulus K (2015). Mechanism of foreign DNA recognition by a CRISPR RNA-guided surveillance complex from Pseudomonas aeruginosa. Nucleic Acids Res.

[CR99] Ron M, Kajala K, Pauluzzi G (2014). Hairy root transformation using Agrobacterium rhizogenes as a tool for exploring cell type-specific gene expression and function using tomato as a model. Plant Physiol.

[CR100] Rui L, Wang L, Chen L, Zhao R (2018) Reduction of tomato-plant chilling tolerance by CRISPR− Cas9-mediated SlCBF1 mutagenesis. 10.1021/acs.jafc.8b02177 10.1021/acs.jafc.8b02177 30096237

[CR101] Santillán Martínez MI, Bracuto V, Koseoglou E (2020). CRISPR/Cas9-targeted mutagenesis of the tomato susceptibility gene PMR4 for resistance against powdery mildew. BMC Plant Biol.

[CR102] Schaart JG, van de Wiel CCM, Smulders MJM (2021). Genome editing of polyploid crops: prospects, achievements and bottlenecks. Transgenic Res.

[CR103] Schulman AH, Oksman-Caldentey KM, Teeri TH (2020). European court of justice delivers no justice to Europe on genome-edited crops. Plant Biotechnol J.

[CR104] Semenova E, Jore MM, Datsenko KA (2011). Interference by clustered regularly interspaced short palindromic repeat (CRISPR) RNA is governed by a seed sequence. Proc Natl Acad Sci USA.

[CR105] Shimatani Z, Kashojiya S, Takayama M (2017). Targeted base editing in rice and tomato using a CRISPR-Cas9 cytidine deaminase fusion. Nat Publ Gr.

[CR106] Shmakov S, Aaron S, Scott D (2017). Diversity and evolution of class 2 CRISPR–Cas systems. Nat Rev Microbiol.

[CR107] Shu P, Li Z, Min D (2020). CRISPR/Cas9-mediated SlMYC2 Mutagenesis adverse to tomato plant growth and MeJA-induced fruit resistance to Botrytis cinerea. J Agric Food Chem.

[CR108] Smargon AA, Cox DBT, Pyzocha NK et al (2017) HHS Public Access 65:618–630. 10.1016/j.molcel.2016.12.023.Cas13b

[CR109] Sorek R, Lawrence CM, Wiedenheft B (2013). CRISPR-mediated adaptive immune systems in bacteria and archaea. Annu Rev Biochem.

[CR110] Soria E (2005) Intervening sequences of regularly spaced prokaryotic repeats derive from foreign genetic elements 174–182. 10.1007/s00239-004-0046-3 10.1007/s00239-004-0046-3 15791728

[CR111] Soyk S, Müller NA, Park SJ (2017). Variation in the flowering gene SELF PRUNING 5G promotes day-neutrality and early yield in tomato. Nat Genet.

[CR112] Springer NM (2013). Epigenetics and crop improvement. Trends Genet.

[CR113] Staals RHJ, Zhu Y, Taylor DW (2014). RNA targeting by the Type III-A CRISPR-Cas Csm complex of Thermus thermophilus. Mol Cell.

[CR114] Stelpflug SC, Eichten SR, Hermanson PJ (2014). Consistent and heritable alterations of DNA methylation are induced by tissue culture in maize. Genetics.

[CR115] Stroud H, Ding B, Simon SA (2013). Plants regenerated from tissue culture contain stable epigenome changes in rice. Elife.

[CR116] Sukrit S, Georg M, Sidote DJ et al (2016) Direct CRISPR spacer acquisition from RNA by a natural reverse-transcriptase-Cas1 fusion protein 351:. 10.1126/science.aad4234.Direct 10.1126/science.aad4234 PMC4898656 26917774

[CR117] Takayama M, Ezura H (2015). How and why does tomato accumulate a large amount of GABA in the fruit?. Front Plant Sci.

[CR118] Takeuchi N, Wolf YI, Makarova KS, Koonin EV (2012). Nature and intensity of selection pressure on crispr-associated genes. J Bacteriol.

[CR119] Tashkandi M, Ali Z, Aljedaani F (2018). Engineering resistance against Tomato yellow leaf curl virus via the CRISPR/Cas9 system in tomato. Plant Signal Behav.

[CR120] Paula de Toledo Thomazella D, Brail Q, Dahlbeck D, Staskawicz B (2016) CRISPR-Cas9 mediated mutagenesis of a DMR6 ortholog in tomato confers broad-spectrum disease resistance. bioRxiv 064824. 10.1101/064824 10.1073/pnas.2026152118 PMC8271637 34215692

[CR121] Ueta R, Abe C, Watanabe T (2017). Rapid breeding of parthenocarpic tomato plants using CRISPR/Cas9. Sci Rep.

[CR122] Ueta R, Abe C, Watanabe T (2017). Rapid breeding of parthenocarpic tomato plants using CRISPR/Cas9. Sci Rep.

[CR123] Van Der Oost J, Westra ER, Jackson RN, Wiedenheft B (2014). Unravelling the structural and mechanistic basis of CRISPR-Cas systems. Nat Rev Microbiol.

[CR124] Van Oosten MJ, Bressan RA, Zhu JK (2014). The role of the epigenome in gene expression control and the epimark changes in response to the environment. CRC Crit Rev Plant Sci.

[CR125] Wang H, Jones B, Li Z (2005). The tomato Aux/IAA transcription factor IAA9 is involved in fruit development and leaf morphogenesis. Plant Cell.

[CR126] Wang L, Chen L, Li R (2017). Reduced drought tolerance by CRISPR/Cas9-mediated SlMAPK3 mutagenesis in tomato plants. J Agric Food Chem.

[CR127] Wang M, Mao Y, Lu Y (2017). Multiplex gene editing in rice using the CRISPR-Cpf1 system. Mol Plant.

[CR128] Wang T, Wei JJ, Sabatini DM, Lander ES (2014) Genetic screens in human cells using the CRISPR-Cas9 system. Science 343(80):80–84. 10.1126/science.1246981 10.1126/science.1246981 PMC3972032 24336569

[CR129] Wang T, Deng Z, Zhang X, et al (2018) Tomato DCL2b is required for the biosynthesis of 22-nt small RNAs, the resulting secondary siRNAs, and the host defense against ToMV. Hortic Res 5. 10.1038/s41438-018-0073-7 10.1038/s41438-018-0073-7 PMC6119189 30181890

[CR130] Westra ER, van Erp PBG, Künne T (2012). CRISPR immunity relies on the consecutive binding and degradation of negatively supercoiled invader DNA by Cascade and Cas3. Mol Cell.

[CR131] Xiao Y, Luo M, Hayes RP (2017). Structure basis for directional R-loop formation and substrate handover mechanisms in Type I CRISPR-Cas system. Cell.

[CR132] Xiong JS, Ding J, Li Y (2015). Genome-editing technologies and their potential application in horticultural crop breeding. Hortic Res.

[CR133] Xu C, Park SJ, Van Eck J, Lippman ZB (2016). Control of inflorescence architecture in tomato by BTB/POZ transcriptional regulators. Genes Dev.

[CR134] Xu R, Qin R, Li H (2019). Enhanced genome editing in rice using single transcript unit CRISPR-LbCpf1 systems. Plant Biotechnol J.

[CR135] Yan L, Wei S, Wu Y (2015). High-Efficiency genome editing in Arabidopsis using YAO promoter-driven CRISPR/Cas9 system. Mol Plant.

[CR136] Yan WX, Hunnewell P, Alfonse LE et al (2019) Functionally diverse type V CRISPR-Cas systems. Science 363(80):88–91. 10.1126/science.aav7271 10.1126/science.aav7271 PMC11258546 30523077

[CR138] Yang H, Gao P, Rajashankar kR, Patel DJ (2016). PAM-dependent target DNA recognition and cleavage by C2c1 CRISPR-Cas endonuclease. Cell.

[CR137] Yang Y, Zhu G, Li R (2017). The RNA editing factor SLORRM4 is required for normal fruit ripening in tomato1. Plant Physiol.

[CR139] Yin Y, Qin K, Song X (2018). BZR1 transcription factor regulates heat stress tolerance through FERONIA receptor-like kinase-mediated reactive oxygen species signaling in tomato. Plant Cell Physiol.

[CR140] Yu QH, Wang B, Li N (2017). CRISPR/Cas9-induced targeted mutagenesis and gene replacement to generate long-shelf life tomato lines. Sci Rep.

[CR141] Zaman QU, Li C, Cheng H, Hu Q (2019). Genome editing opens a new era of genetic improvement in polyploid crops. Crop J.

[CR142] Zeilmaker T, Ludwig NR, Elberse J (2015). Downy mildew resistant 6 and DMR6-like oxygenase 1 are partially redundant but distinct suppressors of immunity in Arabidopsis. Plant J.

[CR143] Zetsche B, Gootenberg JS, Abudayyeh OO (2015). Cpf1 Is a single RNA-guided endonuclease of a Class 2 CRISPR-Cas system. Cell.

[CR144] Zhang J, Rouillon C, Kerou M (2012). Structure and mechanism of the CMR complex for CRISPR-mediated antiviral immunity. Mol Cell.

[CR145] Zhang H, Zhang J, Wei P (2014). The CRISPR/Cas9 system produces specific and homozygous targeted gene editing in rice in one generation. Plant Biotechnol J.

[CR146] Zhang S, Wang L, Zhao R (2018). Knockout of SlMAPK3 reduced disease resistance to Botrytis cinerea in tomato plants. J Agric Food Chem.

[CR147] Zong Y, Wang Y, Li C (2017). Precise base editing in rice, wheat and maize with a Cas9-cytidine deaminase fusion. Nat Biotechnol.

[CR148] Zsögön A, Čermák T, Naves ER (2018). De novo domestication of wild tomato using genome editing. Nat Biotechnol.

